# Feedback Approach for the Relay Channel with Noisy Feedback and Its Security Analysis

**DOI:** 10.3390/e26080651

**Published:** 2024-07-30

**Authors:** Rong Hu, Haonan Zhang, Huan Yang

**Affiliations:** 1 School of Intelligence Technology, Geely University of China, Chengdu 610000, China; hurong@guc.edu.cn; 2 School of Information Science and Technology, Southwest JiaoTong University, Chengdu 610031, China; yanghuan@my.swjtu.edu.cn

**Keywords:** feedback coding, relay channel, noisy feedback, wiretap channel

## Abstract

Relay channels capture the essence of several important communication scenarios such as sensor network and satellite communication. In this paper, first, we propose an efficient coding scheme for an additive white Gaussian noise (AWGN) relay channel in the presence of AWGN feedback, which generalizes the conventional scheme for the AWGN relay channel with noiseless feedback by introducing a lattice-based strategy to eliminate the impact of the feedback channel noise on the performance of the original scheme. Next, we further extend the proposed scheme to the multi-input single-output (MISO) fading relay channel (FRC) with noisy feedback. The key to this extension is to use a pre-coding strategy to transform the MISO channel into a single-input single-output (SISO) channel and applying a two-dimensional lattice coding strategy to deal with the feedback fading channel noise. Finally, we analyze the security performance of our proposed scheme in several cases, and the results of this paper are further illustrated by numerical examples.

## 1. Introduction

The relay channel (RC), which captures the essence of satellite communication [1,2,3], receives much attention in the literature. To explore the fundamental limit of the transmission over the RC, in recent years, the studies [4,5,6,7,8,9,10], respectively, have extended the *decode-and-forward* (DF) strategy [11], the *compress-and-forward* (CF) strategy [11], and the *amplify-and-forward* (AF) strategy [12] for the single relay case to the RC. These extensions have provided corresponding lower bounds on the capacity of the RC according to these relay strategies.

Nowadays, ultra-reliable and low-latency communication (URLLC) [13] serves as one of the key technologies in fifth-generation (5G) wireless communications since it supports many critical services in 5G that require a high level of reliability and low latency. Recently, the study of URLLC in the RC has received much attention, which mainly focuses on performance analysis [14,15,16] and emerging wireless access techniques [17,18,19], evaluating key metrics such as transmission rates, latency, and reliability to improve RC performance. However, there is a notable lack of attention to the design of efficient, low-complexity coding schemes for RC. This gap highlights the need for further exploration into coding strategies that can reduce computational demands while maintaining or improving system performance.

Though channel feedback does not increase the memoryless channel capacity [20], it helps to construct an efficient coding scheme, such as the well-known Schalkwijk–Kailath (SK) scheme [21]. It has been shown that the SK scheme achieves a desired decoding error probability with extremely short coding blocklength [22], which indicates that it may be a good code for URLLC scenarios. Recently, ref. [23] extended the classical SK scheme [21] to the Gaussian relay channel (GRC) with noiseless feedback. In [23], the AF relay strategy and the classical SK coding scheme are combined, and to eliminate the impact of the relay noise on the performance of the original coding scheme, an auxiliary signal is generated, and the difference between the receiver’s received signal and this auxiliary signal is used for the receiver’s decoding. However, note that this SK-type scheme is based on noiseless feedback, which is unrealistic in practical communication scenarios. Then, it is natural to ask: can we extend the SK-type scheme in [23] to the noisy feedback case?

On the other hand, due to the broadcast nature of wireless communication, in recent years, physical layer security (PLS), which started from [24], has been proven to be a useful tool to deal with the eavesdropping problem in wireless communication. In [25], it has already been shown that the classical SK [21] scheme achieves perfect weak secrecy by itself, where the perfect weak secrecy is known as one standard for the PLS. This self-secure property has been extensively studied in the literature; see [26,27,28]. However, we should note that this property holds for the noiseless feedback case, and whether it also holds for the noisy feedback case remains unknown.

To answer the aforementioned questions, in this paper, we aim to extend the SK-type scheme to the GRC with noiseless feedback [23] to the wireless scenario and explore its security performance when an external eavesdropper is considered. First, we propose such an extended scheme for the additive white Gaussian noise (AWGN) RC in the presence of AWGN feedback, where the modulo-lattice function is introduced to mitigate the influence of noise within the feedback channel on the efficacy of the SK scheme.

Furthermore, the utilization of multiple-input, single-output (MISO) technology in satellite relay communications offers significant advantages in enhancing the performance and reliability of the communication system [2,3]. The above scenario can be modeled as using multiple transmitting antennas at the satellite and a single receiving antenna at the ground station and relay station. Then, another question is whether we can extend the scheme for the AWGN RC with AWGN feedback to the MISO fading relay channel (FRC) with noisy feedback. To this end, we further extend the proposed scheme to the MISO fading relay channel (FRC) with noisy feedback. In this extension scheme, to deal with the complex signals in the fading channel, we introduce the two-dimensional complex plane mapping method. Furthermore, the pre-coding strategy is used to transform the feedforward MISO channel into a complex-valued SISO channel, and the beamforming strategy is employed to convert the feedback single-input, multi-output (SIMO) channel into a complex-valued SISO channel. Then, following the scheme for the AWGN RC with AWGN feedback, the scheme for the MISO FRC with noisy feedback is obtained. Finally, we show that our extended scheme may achieve the PLS requirement for some cases.

The remainder of this paper is organized as follows. A formal definition of the RC with noisy feedback is introduced in Section 2. The proposed SK-type scheme for the AWGN RC with AWGN feedback is shown in Section 3. The extended SK-type scheme for the MISO fading RC with noisy feedback and its secrecy analysis are shown in Section 4. Numerical results are given in Section 5. Section 6 summarizes all results in this paper and discusses future works.

## 2. Model Formulation

*The notations*: R and C represent the real and complex space, respectively. The superscript ${\left(\cdot \right)}^{H}$ denotes the conjugate transpose. $\left|\cdot \right|$ represents the absolute value if applied to a complex number or the cardinality if applied to a set. And $∥\cdot ∥$ represents the $l_{2}$-norm of a vector. Unif[a,b] denotes uniform distribution in [a,b]. $N(0,\sigma^{2})$ and $\mathrm{CN}(0,\sigma^{2})$, respectively, denote the Gaussian distribution and circularly symmetric complex Gaussian distribution with mean 0 and covariance $\sigma^{2}$.$Y^{N}=\left(Y_{1},Y_{2},\cdots ,Y_{N}\right)$. $E\left(\cdot \right)$ and $\mathrm{Var}\left(\cdot \right)$ represent statistical expectation and variance for random variables, respectively. The Gaussian Q-function is defined by $Q\left(x\right)=\frac{1}{\sqrt{2\pi }}\int_{x}^{\infty }e^{-\frac{t^{2}}{2}}dt$, and $Q^{-1}\left(\cdot \right)$ is its inverse function. The log function takes base 2 in this paper.

### 2.1. The Gaussian Relay Channel with Noisy Feedback

**Figure 1:**
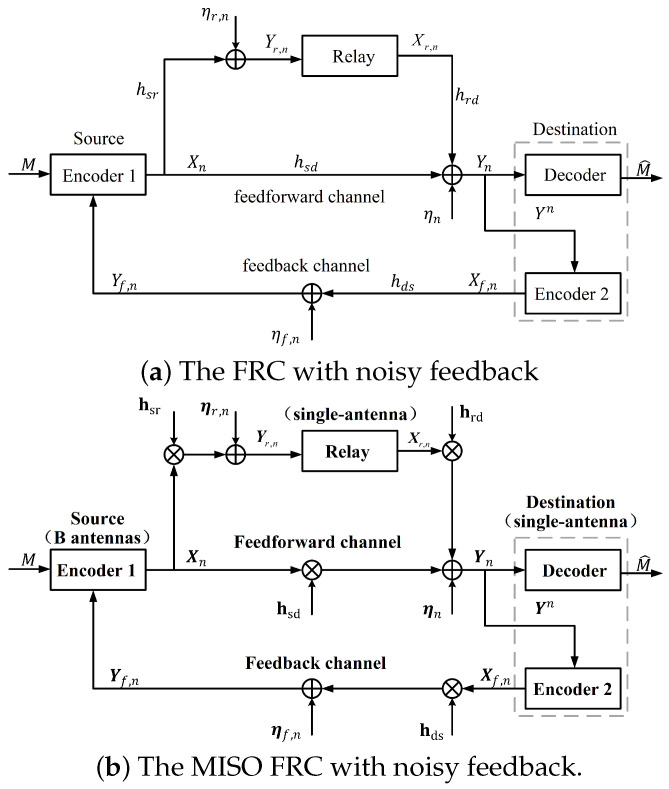
The information-theoretic schematic diagram.

In this subsection, the communication scenario is depicted in Figure 1a, which consists of a Source, a Relay, and a Destination. Source wishes to transmit the message to Destination with the help of Relay, and the message received by Destination can be sent to Source through an AWGN feedback channel. The channel gains of the Source–Relay channel, the Relay–Destination channel, the Source–Destination channel and the Destination–Source channel are denoted by $h_{sr},h_{rd},h_{sd},h_{ds}$, respectively.

So the channel inputs-outputs relationships are given by

(1) $$\begin{array}{cc} \; & \mathrm{Relay}:\;\;\;\;\;Y_{r,n}=h_{sr}X_{n}+\eta_{r,n},\;\;\;\;1\leq n\leq N,\\ \; & \mathrm{Destination}:\;\;\;\;\;Y_{n}=h_{sd}X_{n}+h_{rd}X_{r,n}+\eta_{n},\;\;\;\;1\leq n\leq N,\\ \; & \mathrm{Source}:\;\;\;\;\;Y_{f,n}=h_{ds}X_{f,n}+\eta_{f,n},\;\;\;\;1\leq n\leq N-1. \end{array}$$

where $X_{n}$ (resp. $Y_{n}$) and $X_{r,n}$ (resp. $Y_{r,n}$) are the feedforward inputs (resp. outputs), and $X_{f,n}$ and $Y_{f,n}$ are the feedback channel input and output, respectively. The channel noises of $\eta_{n},\eta_{r,n},\eta_{f,n}$ are independent and identically distributed (i.i.d.) as $N\left(0,\sigma^{2}\right)$, $N\left(0,\sigma_{r}^{2}\right)$, $N\left(0,\sigma_{f}^{2}\right)$, respectively.

**Definition** **1.** *For the GRC with noisy feedback in Figure 1a, a ($N,|M|,P,P_{r},P_{f}$)-code with the average power constraints consist of:*

- *A uniformly distributed message M, which takes values over the set* $M=\left\{1,2,\ldots ,\left|M\right|\right\}$.
- *Encoder 1 with output* $X_{n}=f_{1,n}\left(M,Y_{f}^{n-1}\right)$*, where* $f_{1,n}\left(\cdot \right)$ *is an encoding function of Source at the time index $n\;\left(1\leq n\leq N\right)$, satisfying the average power constraint* $\sum_{n=1}^{N}E\left[X_{n}^{2}\right]\leq NP$.
- *Relay with output* $X_{r,n}=f_{r,n}\left(Y_{r}^{n-1}\right)$*, where* $f_{r,n}\left(\cdot \right)$* is an encoding function of Relay at time index $n\;\left(1\leq n\leq N\right)$, satisfying the average power constraint* $\sum_{n=1}^{N}E\left[X_{r,n}^{2}\right]\leq NP_{r}$.
- *Encoder 2 with output* $X_{f,n}=f_{2,n}\left(Y^{n}\right)$*, where* $f_{2,n}\left(\cdot \right)$*is an encoding function of Destination at time index $n\;\left(1\leq n\leq N-1\right)$, satisfying the average feedback power constraint* $\sum_{n=1}^{N-1}E\left[X_{f,n}^{2}\right]\leq \left(N-1\right)P_{f}$.
- *Decoder has output* $\hat{M}=\psi \left(Y^{N}\right)$*, where ψ is the decoding function of Destination. The average decoding error probability is defined as*
  (2) $$P_{e}=\frac{1}{\left|M\right|}\sum_{m\in M}\mathrm{Pr}\left\{\psi \left(Y^{N}\right)\neq m|m\;\mathrm{was}\;\mathrm{sent}\right\}.$$

**Definition** **2.** *The rate R is said to be $\left(N,\epsilon \right)$-achievable if for given coding block length N and decoding error probability ϵ, there exists a* $\left(N,|M|,P,P_{r},P_{f}\right)$ *-code described in Definition 1 such that*

(3) $$\frac{\log\left|M\right|}{N}\geq R-\epsilon ,\;P_{e}\leq \epsilon .$$

*The*$\left(N,\epsilon \right)$*-capacity [29] of the GRC with noisy feedback is defined as the supremum over all achievable rates in Definition 2, denoted as* $C_{\mathrm{GRC}}^{\mathrm{NFb}}\left(N,\epsilon \right)$.

**Definition** **3.** *The modulo-d function [30,31] (also named the one-dimensional modulo-lattice function) is defined as*

(4) $$M_{d}\left[x\right]=x-d\cdot \left\lfloor \frac{y}{d}+\frac{1}{2}\right\rfloor ,$$

*where d>0 is the step size of the one-dimensional modulo-lattice and $\left\lfloor x\right\rfloor$ represents the largest integer that is smaller than x. Proposition 1 provides the properties of the modulo-d function [30,31].*

**Proposition** **1** ([30,31])**.**

*(1)*  *$M_{d}\left[x\right]\in \left[-\frac{d}{2},\frac{d}{2}\right)$.*
*(2)*  *$M_{d}\left[M_{d}\left[x\right]+y\right]=M_{d}\left[x+y\right]$.*
*(3)*  *if $x+y\in [-\frac{d}{2},\frac{d}{2})$,*
  (5) $$M_{d}\left[x+y\right]=x+y,$$
  *otherwise, a random error termed a·d is added to the right-hand side of (5) for a≠0, which means that a modulo aliasing error has occurred.*
*(4)*  *Let $\nu ∼\mathrm{Unif}[-\frac{d}{2},\frac{d}{2})$, $M_{d}\left[x+y\right]∼\mathrm{Unif}\left[-\frac{d}{2},\frac{d}{2}\right)$ for any x∈R and $E\left[{\left(M_{d}\left[x+\nu \right]\right)}^{2}\right]=d^{2}/12$.*
*(5)*  *For any random variable Y that is statistically independent of ν, $M_{d}\left[Y+\nu \right]$ is statistically independent of Y.*

### 2.2. The MISO Fading Relay Channel with Noisy Feedback

The MISO FRC with noisy feedback studied in this subsection is also depicted in Figure 1b, which consists of a Source equipped with B(B>1) antennas, a Relay, and a Destination with a single-antenna, respectively. The channel gains of the Source-=Relay channel, the Relay-=Destination channel, the Source=-Destination channel and the Destination-=Source channel are denoted by $h_{\mathrm{sr}}\in C^{1\times B},h_{rd}\in C^{1\times 1},h_{\mathrm{sd}}\in C^{1\times B},h_{\mathrm{ds}}\in C^{B\times 1}$, respectively. In this work, the channels of these four links are independent and experience quasi-static fading; i.e., the channel state of each link is constant during one block and varies independently for the next.

So the channel input–output relationships are given by

(6) $$\begin{array}{cc} \; & \mathrm{Relay}:\;\;\;\;\;Y_{r,n}=h_{\mathrm{sr}}X_{n}+\eta_{r,n}\;\;,\;\;\;\;1\leq n\leq N,\\ \; & \mathrm{Destination}:\;\;\;\;\;Y_{n}=h_{\mathrm{sd}}X_{n}+h_{rd}X_{r,n}+\eta_{n}\;\;,\;\;\;\;1\leq n\leq N,\\ \; & \mathrm{Source}:\;\;\;\;\;Y_{f,n}=h_{\mathrm{ds}}X_{f,n}+\eta_{f,n}\;\;,\;\;\;\;1\leq n\leq N-1, \end{array}$$

where $X_{n}\in C^{B\times 1}$ (resp. $Y_{n}\in C^{1\times 1}$) and $X_{r,n}\in C^{1\times 1}$ (resp. $Y_{r,n}\in C^{1\times 1}$) are the feedforward inputs (resp. outputs), and $X_{f,n}\in C^{1\times 1}$ and $Y_{f,n}\in C^{B\times 1}$ are the feedback channel input and output, respectively. The channel noises of $\eta_{n}$ and $\eta_{r,n}$ are i.i.d. as $N\left(0,\sigma^{2}\right)$, $N\left(0,\sigma_{r}^{2}\right)$, respectively, and the elements of $\eta_{f,n}\in C^{B\times 1}$ are i.i.d. as $N\left(0,\sigma_{f}^{2}\right)$.

**Definition** **4.** *For the MISO FRC with noisy feedback in Figure 1b, a $\left(N,|M|,P,P_{r},P_{f}\right)$-code with the average power constraints consists of*

- *A uniformly distributed message M, which takes values over the set* $M=\left\{1,2,\ldots ,\left|M\right|\right\}$.
- *Encoder 1 with output $X_{n}=f_{1,n}\left(M,Y_{f}^{n-1}\right)$, where $f_{1,n}\left(\cdot \right)$ is an encoding function of Source at the time index n(1≤n≤N), satisfying the average power constraint* $\sum_{n=1}^{N}E\left[X_{n}^{H}X_{n}\right]\leq NP$.
- *Relay with output $X_{r,n}=f_{r,n}\left(Y_{r}^{n-1}\right)$, where $f_{r,n}\left(\cdot \right)$ is an encoding function of Relay at time index n(1≤n≤N), satisfying the average power constraint* $\sum_{n=1}^{N}E[|X_{r,n}{|}^{2}]\leq NP_{r}$.
- *Encoder 2 with output $X_{f,n}=f_{2,n}\left(Y^{n}\right)$, where $f_{2,n}\left(\cdot \right)$ is an encoding function of Destination at time index n(1≤n≤N−1), satisfying the average feedback power constraint* $\sum_{n=1}^{N-1}E[|X_{f,n}{|}^{2}]\leq \left(N-1\right)P_{f}$.
- *Decoder has output $\hat{M}=\psi \left(Y^{N}\right)$, where ψ is the decoding function of Destination. The average decoding error probability is also defined as (2).*

**Definition** **5.** *The rate R is said to be $\left(N,\epsilon \right)$-achievable if for given coding blocklength N and decoding error probability ϵ, there exists a* $\left(N,|M|,P,P_{r},P_{f}\right)$*-code described in Definition 4 such that (3). The (N,ϵ)-capacity [29] of the FRC with noisy feedback is defined as the supremum over all achievable rates in Definition 4, denoted as* $C_{\mathrm{FRC}}^{\mathrm{NFb}}\left(N,\epsilon \right)$.

**Definition** **6.** *The two-dimensional modulo-lattice function [31] (modulo-*Λ* function) is defined as*

(7) $$M_{\Lambda }\left[X\right]\overset{\mathrm{def}}{=}X-Q_{\Lambda }\left[X\right],$$

*where $\Lambda =\Lambda_{R}+j\Lambda_{I}$ is a complex plane with $\Lambda \in [\pm \frac{d}{2},\pm j\frac{d}{2})$, d>0, j is the imaginary unit, X is a complex-valued number, and $Q_{\Lambda }\left[X\right]$ is the nearest neighbor quantization of X with respect to *Λ*.*

**Proposition** **2** ([31])**.**

*(1)*  *The distributive law $M_{\Lambda }\left[M_{\Lambda }\left[X\right]+Y\right]=M_{\Lambda }\left[X+Y\right]$.*
*(2)*  *If X+Y∈Λ, $M_{\Lambda }\left[X+Y\right]=X+Y$; otherwise, a modulo-aliasing error has occurred.*
*(3)*  *Let the dither signal V be uniform over *Λ*; then, $M_{\Lambda }\left[X+V\right]$ is uniform over Λ, and $\mathrm{Var}\left(M_{\Lambda }\left[X+V\right]\right)=d^{2}/12+d^{2}/12=d^{2}/6$.*
*(4)*  *For any random variable X that is statistically independent of the dither signal V, $M_{\Lambda }\left[X+V\right]$ is statistically independent of X.*

## 3. An SK-Type Feedback Scheme for the Gaussian Relay Channel with Noisy Feedback

In this section, an SK-type feedback scheme for the Gaussian relay channel with noisy feedback is proposed. The key to this scheme involves the introduction of the modulo-lattice function to mitigate the impact of feedback channel noise on the performance of the SK-type scheme. Additionally, an auxiliary signal is generated at the receiver, which is the receiver’s estimation of the output of the AF strategy-based relay node, and using a difference between the received signal and this auxiliary signal to do the receiver’s MMSE about the transmitted message. The main result and details about this scheme are given below.

### 3.1. Main Result

**Theorem** **1.** *For given coding blocklength N and decoding error probability ϵ, the lower bound on the* $\left(N,\epsilon \right)$ *-capacity* $C_{\mathrm{GRC}}^{\mathrm{NFb}}\left(N,\epsilon \right)$ *of the GRC with noisy feedback is given by*

(8) $$C_{\mathrm{GRC}}^{\mathrm{NFb}}\left(N,\epsilon \right)\geq R_{\mathrm{GRC}}^{\mathrm{NFb}}\left(N,\epsilon \right)=R_{\mathrm{GRC}}^{*}\left(N,\epsilon \right)-R_{\mathrm{GRC}}^{◊}\left(N,\epsilon \right),$$

*where*

(9) $$R_{\mathrm{GRC}}^{*}\left(N,\epsilon \right)=\frac{1}{2}\log\left(1+\frac{{\left(h_{sd}\sqrt{\frac{P}{\phi }}+h_{sr}h_{rd}\beta_{↑}\sqrt{P}\rho_{↑}^{*}\right)}^{2}}{\Gamma_{↑}}\right),$$

(10) $$R_{\mathrm{GRC}}^{◊}\left(N,\epsilon \right)=\frac{1}{N}\left(2R_{\mathrm{GRC}}^{*}\left(N,\epsilon \right)+\log\mu \right),$$

(11) $$\phi =\frac{P_{f}h_{ds}^{2}}{P_{f}h_{ds}^{2}-L_{↑}\sigma_{f}^{2}},$$

(12) $$L_{↑}=\frac{1}{3}{\left(Q^{-1}\left(\frac{\epsilon }{4\left(N-1\right)}\right)\right)}^{2},$$

(13) $$\beta_{↑}=\sqrt{\frac{P_{r}}{h_{sr}^{2}P+\sigma_{r}^{2}}},$$

(14) $$\mu =2Q^{-1}\left(\frac{\epsilon }{4}\right)\sqrt{\frac{\sigma^{2}}{12Ph_{sd}^{2}}{\left(1+\frac{h_{sd}^{2}\frac{P}{\phi }}{h_{sd}^{2}P\left(1-\frac{1}{\phi }\right)+\sigma^{2}}\right)}^{-1}},$$

(15) $$\Gamma_{↑}=h_{sr}^{2}h_{rd}^{2}\beta_{↑}^{2}P\left(\left(1-\frac{1}{\phi }\right)-{\left(1-\frac{1}{A_{↑}\left(\rho_{↑}^{*}\right)}\right)}^{2}{\left(\rho_{↑}^{*}\right)}^{2}\right)+F_{↑},$$

(16) $$F_{↑}=h_{sd}^{2}P\left(1-\frac{1}{\phi }\right)+h_{rd}^{2}\beta_{↑}^{2}\sigma_{r}^{2}+\sigma^{2},$$

and the correlation coefficient $\rho_{↑}^{*}$ is given by the unique solution in [0, 1] of the following equation:

(17) $$\begin{array}{cc} \; & \rho_{↑}^{2}=\frac{{\left(A_{↑}\left(\rho_{↑}\right)\right)}^{2}}{\phi }\times \\ \; & {\left(1+\frac{{\left(h_{sd}\sqrt{\frac{P}{\phi }}+h_{sr}h_{rd}\beta_{↑}\sqrt{P}\rho_{↑}\right)}^{2}}{h_{sr}^{2}h_{rd}^{2}\beta_{↑}^{2}P\left(\left(1-\frac{1}{\phi }\right)-{\left(1-\frac{1}{A_{↑}\left(\rho_{↑}\right)}\right)}^{2}\rho_{↑}^{2}\right)+F_{↑}}\right)}^{-1}, \end{array}$$

(18) $$\begin{array}{cc} \; & A_{↑}\left(\rho_{↑}\right)=1+\\ \; & \frac{Ph_{sd}\left(h_{sd}\sqrt{\frac{1}{\phi }}+h_{sr}h_{rd}\beta_{↑}\rho_{↑}\right)\left(\sqrt{\frac{1}{\phi }}-\sqrt{\phi }\right)}{h_{sr}^{2}h_{rd}^{2}\beta_{↑}^{2}P\left(\left(1-\frac{1}{\phi }\right)-{\left(1-\frac{1}{A_{↑}\left(\rho_{↑}\right)}\right)}^{2}\rho_{↑}^{2}\right)+F_{↑}}. \end{array}$$

**Remark** **1.** *When the blocklength N is sufficiently large, the achievable rate* $R_{\mathrm{GRC}}^{\mathrm{NFb}}\left(N,\epsilon \right)$ *approaches* $R_{\mathrm{GRC}}^{*}\left(N,\epsilon \right)$*, which is given in (9).*

**Remark** **2.** *The upper bound* $R_{\mathrm{GRC}}^{\mathrm{PFb}}$ *of the GRC with noisy feedback is given in [23], where*

(19) $$R_{\mathrm{GRC}}^{\mathrm{PFb}}=\frac{1}{2}\log\left(1+\frac{P{\left(h_{sd}+h_{sr}h_{rd}\beta_{↑}\rho_{↑}^{*}\right)}^{2}}{h_{rd}^{2}\beta_{↑}^{2}\sigma_{r}^{2}+\sigma^{2}}\right),$$

$\rho_{↑}^{*}$ is the unique solution in $\left[0,1\right]$ of the following equation

(20) $$\rho_{↑}^{2}={\left(1+\frac{P{\left(h_{sd}+h_{sr}h_{rd}\beta_{↑}\rho_{↑}\right)}^{2}}{h_{rd}^{2}\beta^{2}\sigma_{r}^{2}+\sigma^{2}}\right)}^{-1}.$$

*For the GRC with noisy feedback, letting the power of the feedback channel $P_{f}$ be sufficiently large, from (11), (15) and (18), we conclude that*

(21) $$\lim_{P_{f}\to \infty }\phi =1,\lim_{P_{f}\to \infty }A_{↑}\left(\rho_{↑}\right)=1,\lim_{P_{f}\to \infty }\Gamma_{↑}=h_{rd}^{2}\beta_{↑}^{2}\sigma_{r}^{2}\;+\;\sigma^{2}.$$

*Substituting (21) into (17), we conclude that (17) can be rewritten as (20). Combined with Remark 1, as N increases,* $R_{\mathrm{GRC}}^{\mathrm{NFb}}\left(N,\epsilon \right)$ *in Theorem 1 approaches* $R_{\mathrm{GRC}}^{\mathrm{PFb}}$ *in (19).*

### 3.2. Our Achievable Scheme

#### 3.2.1. Message Mapping Method

For a given coding blocklength *N* and decoding error probability ϵ, let $\left|M\right|=2^{NR(N,\epsilon )}$, where *M* is the transmitted message uniformly distributed over the set $M=\{1,2,\ldots ,2^{NR(N,\epsilon )}\}$. Divide the interval $\left[-0.5,0.5\right]$ into $2^{NR(N,\epsilon )}$ equally spaced sub-intervals, and the center of each sub-interval is mapped one-to-one to a message value in M. Let θ be the center of each sub-interval with respect to the message *M*. Since *M* is equi-probably distributed over the set M, θ is approximately uniformly distributed over the interval $\left[-0.5,0.5\right]$ (i.e., $\theta ∼\mathrm{Unif}\left[-0.5,0.5\right]$) and has a variance of approximately 1/12, i.e., $E[\theta^{2}]=1/12$.

#### 3.2.2. Dither Signal for the Feedback Channel Power Constaint

In the proposed scheme, we introduce a dither signal $\nu_{n}\left(1\leq n\leq N-1\right)$ to ensure the transmitted codewords satisfy the feedback channel’s power constraint at time *n*. Here $\nu_{n}$ is perfectly known by Source, and it is i.i.d. generated and uniformly distributed over [−d/2,d/2). In addition, $\gamma_{n}\left(1\leq n\leq N-1\right)$ is chosen to avoid modulo-aliasing errors occurring in Source.

#### 3.2.3. Coding Procedure

At time instant 1, Source transmits

(22) $$X_{1}=\sqrt{12P}\theta ,$$

where $E[X_{1}^{2}]=P$, which satisfies the average power constraint of Source.

Meanwhile, Relay remains quiet, and Destination obtains the first output signal

(23) $$Y_{1}=h_{sd}X_{1}+\eta_{1}=h_{sd}\sqrt{12P}\theta +\eta_{1},$$

then computes the first estimation of θ by

(24) $${\hat{\theta }}_{1}=\frac{Y_{1}}{h_{sd}\sqrt{12P}}=\theta +\frac{\eta_{1}}{h_{sd}\sqrt{12P}}≜\theta +\varepsilon_{1},$$

where $\varepsilon_{1}$ is the first estimation error, denoted by

(25) $$\varepsilon_{1}≜{\hat{\theta }}_{1}-\theta =\frac{\eta_{1}}{h_{sd}\sqrt{12P}},$$

and its variance is

(26) $$\alpha_{1}≜\mathrm{Var}\left(\varepsilon_{1}\right)=\frac{\sigma^{2}}{12Ph_{sd}^{2}}.$$

Then, Destination encodes

(27) $$X_{f,1}=M_{d}\left[\gamma_{1}{\hat{\theta }}_{1}+\nu_{1}\right],$$

and sends $X_{f,1}$ back to Source. Here from Property (1) of Proposition 1 and $d=\sqrt{12P_{f}}$, the power constraint of $X_{f,1}$ satisfies $P_{f}$.

Then Source receives the feedback signal

(28) $$Y_{f,1}=h_{ds}X_{f,1}+\eta_{f,1}=h_{ds}M_{d}\left[\gamma_{1}{\hat{\theta }}_{1}+\nu_{1}\right]+\eta_{f,1}.$$

At time 2, by using the feedback signals $Y_{f,1}$ (see (28)), Source firstly computes the noisy versions of the Destination’s estimation errors, i.e.,

(29) $$\begin{array}{cc} \varepsilon_{f,1} & =\frac{1}{\gamma_{1}}M_{d}\left[\frac{1}{h_{ds}}Y_{f,1}-\gamma_{1}\theta -\nu_{1}\right]\\ \; & \overset{\left(a\right)}{=}\frac{1}{\gamma_{1}}M_{d}\left[M_{d}\left[\gamma_{1}{\hat{\theta }}_{1}+\nu_{1}\right]+\frac{\eta_{f,1}}{h_{\mathrm{ds}}}-\gamma_{1}\theta -\nu_{1}\right]\\ \; & \overset{\left(b\right)}{=}\frac{1}{\gamma_{1}}M_{d}\left[\gamma_{1}\varepsilon_{1}+\frac{\eta_{f,1}}{h_{ds}}\right], \end{array}$$

where (a) follows from (28), (b) follows from Property (1) of Proposition 1 and the fact that $\varepsilon_{1}={\hat{\theta }}_{1}-\theta$. In accordance with (4), we know that if $\gamma_{1}\varepsilon_{1}+\eta_{f,1}/h_{ds}\in \left[-d/2,d/2\right)$, (29) can be rewritten by

(30) $$\varepsilon_{f,1}=\frac{1}{\gamma_{1}}M_{d}\left[\gamma_{1}\varepsilon_{1}+\frac{\eta_{f,1}}{h_{ds}}\right]=\varepsilon_{1}+\frac{\eta_{f,1}}{\gamma_{1}h_{ds}},$$

otherwise, it implies that a modulo-aliasing error does occur in Source. Hence understanding how to choose the modulation coefficient $\gamma_{1}$ is crucial to avoid the modulo-aliasing error.

After decoding $\varepsilon_{f,1}$, Source sends

(31) $$X_{2}=\sqrt{\frac{P}{\alpha_{f,1}}}\varepsilon_{f,1},$$

where $\alpha_{f,1}≜\mathrm{Var}\left(\varepsilon_{f,1}\right)$, and $E[X_{2}^{2}]=P$, which satisfies the average power constraint of Source.

At this time instant, Relay also keeps quiet. And once it receives

(32) $$Y_{2}=h_{sd}X_{2}+\eta_{2}=h_{sd}\sqrt{\frac{P}{\alpha_{f,1}}}\varepsilon_{f,1}+\eta_{2},$$

Destination carries out the MMSE of $\varepsilon_{1}$ based on $Y_{2}$ and updates the estimation of θ by computing

(33) $${\hat{\theta }}_{2}={\hat{\theta }}_{1}-\frac{E\left[\varepsilon_{1}Y_{2}\right]}{E\left[Y_{2}^{2}\right]}Y_{2}=\theta +\varepsilon_{1}-\frac{E\left[\varepsilon_{1}Y_{2}\right]}{E\left[Y_{2}^{2}\right]}Y_{2}=\theta +\varepsilon_{2},$$

where the second estimation error $\varepsilon_{2}$ is denoted as

(34) $$\varepsilon_{2}=\varepsilon_{1}-\frac{E\left[\varepsilon_{1}Y_{2}\right]}{E\left[Y_{2}^{2}\right]}Y_{2},$$

and the variance of the estimation error is denoted as

(35) $$\alpha_{2}≜\mathrm{Var}\left(\varepsilon_{2}\right).$$

*Iteration:*

At time instant n−1(3≤n≤N), Destination sends

(36) $$X_{f,n-1}=M_{d}\left[\gamma_{n-1}{\hat{\theta }}_{n-1}+\nu_{n-1}\right]$$

back to Source.

At time n(3≤n≤N), once it receives the feedback signals

(37) $$Y_{f,n-1}=h_{ds}M_{d}\left[\gamma_{n-1}{\hat{\theta }}_{n-1}+\nu_{n-1}\right]+\eta_{n-1},$$

Source computes the noisy version of the Destination’s estimation errors, i.e.,

(38) $$\begin{array}{cc} \varepsilon_{f,n-1} & =\frac{1}{\gamma_{n-1}}M_{d}\left[\frac{1}{h_{ds}}Y_{f,n-1}-\gamma_{n-1}\theta -\nu_{n-1}\right]\\ \; & \overset{\left(a\right)}{=}\frac{1}{\gamma_{n-1}}M_{d}\left[\gamma_{n-1}\varepsilon_{n-1}+\frac{\eta_{f,n-1}}{h_{ds}}\right]\\ \; & \overset{\left(b\right)}{=}\varepsilon_{n-1}+\frac{\eta_{f,n-1}}{\gamma_{n-1}h_{ds}}, \end{array}$$

where (a) follows from (37), Property (1) of Proposition 1 and the fact that $\varepsilon_{n-1}={\hat{\theta }}_{n-1}-\theta$. (b) follows from $\gamma_{n-1}\varepsilon_{n-1}+\eta_{f,n-1}/h_{ds}\in \left[-d/2,d/2\right)$, which means that modulo-aliasing error does not occur in Source.

After decoding $\varepsilon_{f,n-1}$, Source transmits

(39) $$X_{n}=\sqrt{\frac{P}{\alpha_{f,n-1}}}\varepsilon_{f,n-1},$$

where $\alpha_{f,n-1}≜\mathrm{Var}\left(\varepsilon_{f,n-1}\right)$, and $E[X_{n}^{2}]=P$, which satisfies the average power constraint of Source.

At time instant n(3≤n≤N), Relay does not remain quiet and transmits the received messages from Source to Destination through an AF strategy, that is, it transmits a scaled version of its observation in the previous step, i.e.,

(40) $$\begin{array}{cc} X_{r,n} & =\beta Y_{r,n-1}=\beta \left(h_{sr}X_{n-1}+\eta_{r,n-1}\right)\\ \; & =\beta \left(h_{sr}\sqrt{\frac{P}{\alpha_{f,n-2}}}\varepsilon_{f,n-2}+\eta_{r,n-1}\right), \end{array}$$

where the scaling factor is chosen as

(41) $$\beta =\sqrt{\frac{P_{r}}{h_{sr}^{2}P+\sigma_{r}^{2}}},$$

and $E\left[X_{r,n}^{2}\right]=\beta^{2}E\left[Y_{r,n-1}^{2}\right]=P_{r}$, which satisfies the average power constraint of Relay.

So, Destination obtains the *n*-th channel output signal

(42) $$\begin{array}{cc} Y_{n} & =h_{sd}X_{n}+h_{rd}X_{r,n}+\eta_{n}\\ \; & =h_{sd}\sqrt{\frac{P}{\alpha_{f,n-1}}}\varepsilon_{f,n-1}+\beta h_{sr}h_{rd}\sqrt{\frac{P}{\alpha_{f,n-2}}}\varepsilon_{f,n-2}\\ \; & +\beta h_{\mathrm{rd}}\eta_{r,n-1}+\eta_{n}. \end{array}$$

Upon receiving $Y_{n}$, Destination first computes the auxiliary signal, that is,

(43) $${\hat{Y}}_{n}=h_{sr}h_{rd}\beta \sqrt{\frac{P}{\alpha_{f,n-2}}}{\hat{\varepsilon }}_{n-2},$$

and this auxiliary signal ${\hat{Y}}_{n}$ will be used to form the innovation $I_{n}$ based on the fact that $\varepsilon_{n-1}=\varepsilon_{n-2}-{\hat{\varepsilon }}_{n-2}$,

(44) $$\begin{array}{cc} I_{n} & ≜Y_{n}-{\hat{Y}}_{n}\\ \; & =h_{sd}\sqrt{\frac{P}{\alpha_{f,n-1}}}\varepsilon_{f,n-1}+h_{sr}h_{rd}\beta \sqrt{\frac{P}{\alpha_{f,n-2}}}\varepsilon_{n-1}\\ \; & +h_{sr}h_{rd}\beta \sqrt{\frac{P}{\alpha_{f,n-2}}}\frac{\eta_{f,n-2}}{\gamma_{n-2}h_{ds}}+\beta h_{rd}\eta_{r,n-1}+\eta_{n}. \end{array}$$

and it carries out the MMSE of $\epsilon_{n-1}$ based on $I_{n}$, then updates the estimation of θ by computing

(45) $${\hat{\theta }}_{n}={\hat{\theta }}_{n-1}-\frac{E\left[\varepsilon_{n-1}I_{n}\right]}{E\left[I_{n}^{2}\right]}I_{n}=\theta +\varepsilon_{n},$$

where the estimation error of θ is denoted as

(46) $$\varepsilon_{n}=\varepsilon_{n-1}-\frac{E\left[\varepsilon_{n-1}I_{n}\right]}{E\left[I_{n}^{2}\right]}I_{n},$$

and the variance of the estimation error is denoted as

(47) $$\alpha_{n}≜\mathrm{Var}\left(\varepsilon_{n}\right).$$

At time instant *N*, Destination computes the final estimation

(48) $${\hat{\theta }}_{N}=\theta +\varepsilon_{N}.$$

According to the mapping method in Section 3.2.1, if the final estimation error is given by

(49) $$\varepsilon_{N}\in \left[-\frac{0.5}{2^{NR\left(N,\epsilon \right)}},\frac{0.5}{2^{NR\left(N,\epsilon \right)}}\right),$$

the final estimation ${\hat{\theta }}_{N}$ is closest to the transmitted point θ, and thus Destination successfully decodes the transmitted message *M*.

**Figure 2:**
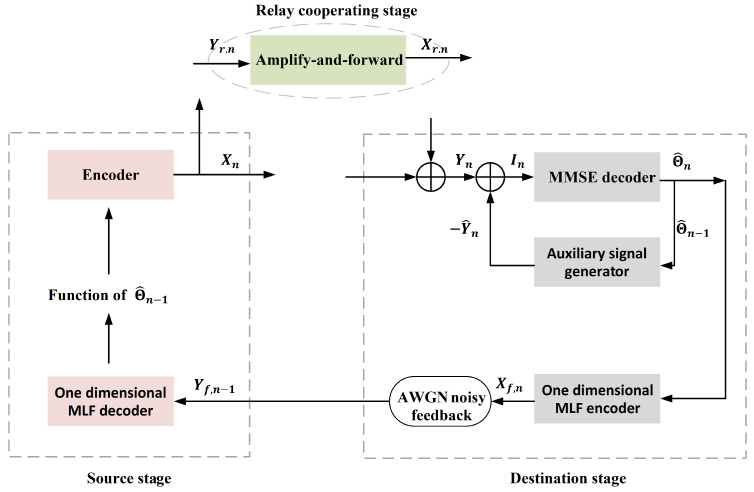
The schematic diagram of the proposed SK-Type finite blocklength (FBL) scheme for the GRC with noisy feedback.

For better understanding, the schematic diagram of the above proposed scheme is shown in Figure 2.

**Proof.**  The detailed proof is given in Appendix A. □

## 4. An SK-Type Scheme for the MISO Fading Relay Channel with Noisy Feedback and Its Security Analysis

In this section, an SK-type scheme for the MISO fading relay channel with noisy feedback is proposed. In this extension scheme, to deal with complex signals in the fading channel, we introduce the two-dimensional complex plane mapping method. Furthermoreavesdropper does not know t, a pre-coding strategy is used to transform the feedforward MISO channel into a complex-valued SISO channel, while a beamforming strategy is used to transform the feedback SIMO channel into a complex-valued SISO channel. Consequently, by following the scheme for the AWGN RC with AWGN feedback, we obtain the scheme for the MISO FRC with noisy feedback. The main result and security analysis of this extension scheme are given below.

### 4.1. Main Result

**Theorem** **2.** *For given coding blocklength N and decoding error probability ϵ, the lower bound on the* $\left(N,\epsilon \right)$*-capacity* $C_{\mathrm{FRC}}^{\mathrm{NFb}}\left(N,\epsilon \right)$ *of the FRC with noisy feedback is given by*

(50) $$C_{\mathrm{FRC}}^{\mathrm{NFb}}\left(N,\epsilon \right)\geq R_{\mathrm{FRC}}^{\mathrm{NFb}}\left(N,\epsilon \right)=R_{\mathrm{FRC}}^{*}\left(N,\epsilon \right)-R_{\mathrm{FRC}}^{◊}\left(N,\epsilon \right),$$

*where*

(51) $$\begin{array}{cc} \; & R_{\mathrm{FRC}}^{*}\left(N,\epsilon \right)\\ = & \frac{1}{2}\log\left(1+\frac{{\left(∥h_{\mathrm{sd}}∥\sqrt{\frac{P}{\Phi }}+∥h_{\mathrm{sr}}∥\cdot \left|h_{rd}\right|\beta \sqrt{P}\rho^{*}\right)}^{2}}{\Gamma }\right), \end{array}$$

(52) $$R_{\mathrm{FRC}}^{◊}\left(N,\epsilon \right)=\frac{1}{N}\left(2R_{\mathrm{FRC}}^{*}\left(N,\epsilon \right)+\log\lambda \right),$$

(53) $$\Phi =\frac{P_{f}{∥h_{\mathrm{ds}}∥}^{2}}{P_{f}{∥h_{\mathrm{ds}}∥}^{2}-L\sigma_{f}^{2}},$$

(54) $$L=\frac{1}{3}{\left(Q^{-1}\left(\frac{\epsilon }{8\left(N-1\right)}\right)\right)}^{2},$$

(55) $$\beta =\sqrt{\frac{P_{r}}{{∥h_{\mathrm{sr}}∥}^{2}P+\sigma_{r}^{2}}},$$

(56) $$\lambda =Q^{-1}\left(\frac{\epsilon }{8}\right)\sqrt{\frac{\sigma^{2}}{3P{∥h_{\mathrm{sd}}∥}^{2}}{\left(1+\frac{{∥h_{\mathrm{sd}}∥}^{2}\frac{P}{\Phi }}{{∥h_{\mathrm{sd}}∥}^{2}P\left(1-\frac{1}{\Phi }\right)+\sigma^{2}}\right)}^{-1}},$$

(57) $$\Gamma ={∥h_{\mathrm{sr}}∥}^{2}{\left|h_{rd}\right|}^{2}\beta^{2}P\left(\left(1-\frac{1}{\Phi }\right)-{\left(1-\frac{1}{A\left(\rho^{*}\right)}\right)}^{2}{\left(\rho^{*}\right)}^{2}\right)+F,$$

(58) $$F={∥h_{\mathrm{sd}}∥}^{2}P\left(1-\frac{1}{\Phi }\right)+{\left|h_{rd}\right|}^{2}\beta^{2}\sigma_{r}^{2}+\sigma^{2},$$

and the correlation coefficient $\rho^{*}$ is given by the unique solution in [0, 1] of the following equation

(59) $$\begin{array}{cc} \; & \rho^{2}=\frac{{\left(A\left(\rho \right)\right)}^{2}}{\Phi }\times \\ \; & {\left(1+\frac{{\left(∥h_{\mathrm{sd}}∥\sqrt{\frac{P}{\Phi }}+∥h_{\mathrm{sr}}∥\cdot \left|h_{rd}\right|\beta \sqrt{P}\rho \right)}^{2}}{{∥h_{\mathrm{sr}}∥}^{2}{\left|h_{rd}\right|}^{2}\beta^{2}P\left(\left(1-\frac{1}{\Phi }\right)-{\left(1-\frac{1}{A\left(\rho \right)}\right)}^{2}\rho^{2}\right)+F}\right)}^{-1}, \end{array}$$

(60) $$\begin{array}{cc} \; & A\left(\rho \right)=1+\\ \; & \frac{P∥h_{\mathrm{sd}}∥\left(∥h_{\mathrm{sd}}∥\sqrt{\frac{1}{\Phi }}+∥h_{\mathrm{sr}}∥\cdot \left|h_{rd}\right|\beta \rho \right)\left(\sqrt{\frac{1}{\Phi }}-\sqrt{\Phi }\right)}{{∥h_{\mathrm{sr}}∥}^{2}{\left|h_{rd}\right|}^{2}\beta^{2}P\left(\left(1-\frac{1}{\Phi }\right)-{\left(1-\frac{1}{A\left(\rho \right)}\right)}^{2}\rho^{2}\right)+F}. \end{array}$$

**Remark** **3.** *When the blocklength N is sufficiently large, the achievable rate* $R_{\mathrm{FRC}}^{\mathrm{NFb}}\left(N,\epsilon \right)$ *approaches* $R_{\mathrm{FRC}}^{*}\left(N,\epsilon \right)$*, which is given in (51).*

**Remark** **4.** *For the MISO FRC without feedback, the AF lower bound* $R_{\mathrm{FRC}}^{\mathrm{AF}}$ *is given by [32], i.e.,*

(61) $$R_{\mathrm{FRC}}^{\mathrm{AF}}=\log\left(1+\frac{{∥h_{\mathrm{sd}}∥}^{2}P}{\sigma^{2}}+\frac{\frac{{∥h_{\mathrm{sr}}∥}^{2}P}{\sigma_{r}^{2}}\cdot \frac{{\left|h_{rd}\right|}^{2}P_{r}}{\sigma^{2}}}{\frac{{∥h_{\mathrm{sr}}∥}^{2}P}{\sigma_{r}^{2}}+\frac{{\left|h_{rd}\right|}^{2}P_{r}}{\sigma^{2}}+1}\right).$$

**Proof.**  The detailed proof is given in Appendix B. □

### 4.2. Security Analysis of the Proposed Extended Scheme

**Figure 3:**
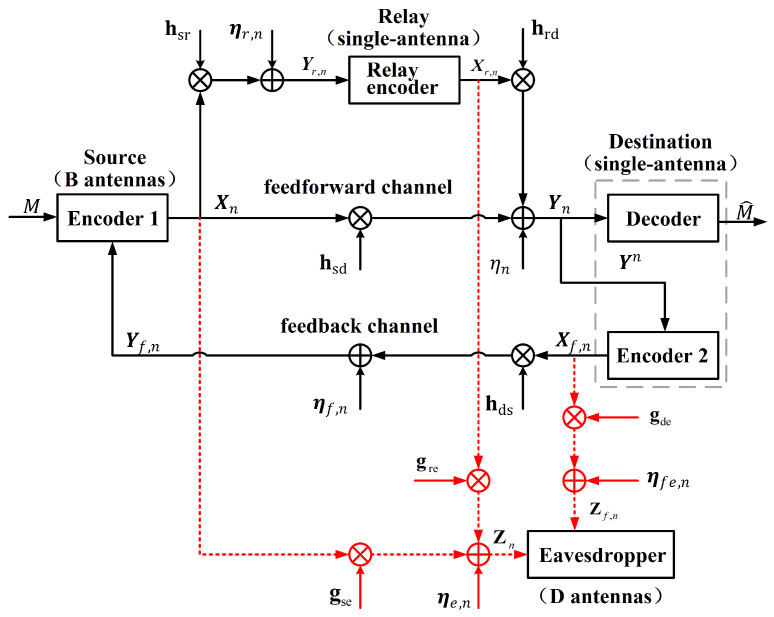
The MISO FRC with noisy feedback and a multi-antenna external eavesdropper.

The communication scenario is similar to Figure 1b, except that the existence of Eavesdropper equipped with B(B>1) antennas. The information-theoretic schematic diagram is depicted in Figure 3. We assume that Eavesdropper can simultaneously eavesdrop on the signals of both the forward and feedback channels. Here, the channel gains of the Source–Eavesdropper channel, the Relay–Eavesdropper channel and the Destination–Eavesdropper channel are, respectively, denoted by $g_{\mathrm{se}}\in C^{D\times B},g_{\mathrm{re}}\in C^{D\times 1},g_{\mathrm{de}}\in C^{D\times 1}$. We assume that these three links are independent of each other and experience quasi-static fading.

So, the signals received by Eavesdropper are given by

(62) $$\begin{array}{cc} \; & Z_{n}=g_{\mathrm{se}}X_{n}+g_{\mathrm{re}}X_{r,n}+\eta_{e,n},1\leq n\leq N,\\ \; & Z_{f,n}=g_{\mathrm{de}}X_{f,n}+\eta_{fe,n},1\leq n\leq N-1, \end{array}$$

where $Z_{n},Z_{f,n}\in C^{D\times 1}$, $X_{n}\in C^{B\times 1},X_{r,n}\in C^{1\times 1},X_{f,n}\in C^{1\times 1}$ represent the input of Source, Relay and Destination, respectively. $\eta_{e,n}∼\mathrm{CN}\left(0,\sigma_{e}^{2}\right),\eta_{fe,n}∼\mathrm{CN}\left(0,\sigma_{fe}^{2}\right)$ are the channel noise, which are i.i.d. across the time index *n*.

**Definition** **7.**  *Following the secrecy criteria defined in [33], a normalized eavesdropper’s equivocation (also called the secrecy level) is adopted in this paper and is given by*

(63) $$\Delta =\frac{H\left(M|Z_{1}^{N},Z_{f,1}^{N-1},h,g\right)}{H\left(M\right)},0\leq \Delta \leq 1,$$

*where $Z_{1}^{N}=\left(Z_{1},Z_{2},\cdots ,Z_{N}\right)$, $Z_{f,1}^{N-1}=\left(Z_{f,1},Z_{f,2},\cdots ,Z_{f,N-1}\right)$, $h=(h_{\mathrm{sr}},h_{rd},h_{\mathrm{sd}},h_{\mathrm{ds}})$, $g=(g_{\mathrm{se}},g_{\mathrm{re}},g_{\mathrm{de}})$. Here, note that Δ=1 corresponds to perfect secrecy, indicating that Eavesdropper cannot obtain any useful information, which was first introduced in [34], and Δ=0 corresponds to no secrecy constraint.*

*Furthermore, in terms of whether Eavesdropper is powerful enough to acquire the complex dither signal sequence $V_{1}^{N-1}$ shared between legal parties in our scheme, we define the following two cases.*

- ***Case I:*** *Eavesdropper does not know the complex dither signal sequence $V_{1}^{N-1}$, and the secrecy level is defined by*
(64) $$\Delta_{I}=\frac{H\left(M|Z_{1}^{N},Z_{f,1}^{N-1},h,g\right)}{H\left(M\right)},0\leq \Delta_{I}\leq 1.$$
- ***Case II:*** *Eavesdropper knows the complex dither signal sequence $V_{1}^{N-1}$, and the secrecy level is defined by*
(65) $$\Delta_{II}=\frac{H\left(M|Z_{1}^{N},Z_{f,1}^{N-1},V_{1}^{N-1},h,g\right)}{H\left(M\right)},0\leq \Delta_{II}\leq 1.$$

**Theorem** **3.** *For given coding blocklength N and decoding error probability ϵ, the secrecy levels $\Delta_{I}$ and $\Delta_{II}$ of the proposed scheme are, respectively, lower-bounded by*

(66) $$\Delta_{I}\geq 1-\frac{\kappa_{1}}{NR_{\mathrm{FRC}}^{\mathrm{NFb}}\left(N,\epsilon \right)},\Delta_{II}\geq 1-\frac{\kappa_{1}+\kappa_{2}}{NR_{\mathrm{FRC}}^{\mathrm{NFb}}\left(N,\epsilon \right)},$$

*where*

(67) $$\begin{array}{cc} \; & \kappa_{1}=log\left(1+\frac{{∥h_{\mathrm{sd}}\cdot g_{\mathrm{se}}^{H}∥}^{2}P}{{∥h_{\mathrm{sd}}∥}^{2}\sigma_{e}^{2}}\right),\\ \; & \kappa_{2}=\left(N-1\right)log\left(1+\frac{{∥g_{\mathrm{de}}∥}^{2}P_{f}}{\sigma_{fe}^{2}}\right), \end{array}$$

*Note that $\kappa_{1}$ and $\kappa_{2}$, respectively, represent the information leaked in Source at time *1* and in the feedback channel from time *1* to N−1.*

**Proof.**  The detailed proof is given in Appendix C. □

## 5. Numerical Results

The aim of these numerical results is to evaluate the performance of our proposed schemes versus different parameters. Here, note that the simulation results for the fading scenario are based on an average of 1000 independent channel realizations.

**Figure 4:**
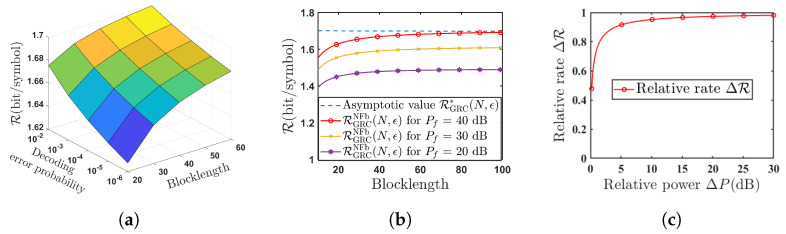
Performance analysis of the proposed scheme for the GRC with noisy feedback ($P_{r}\;=\;10\;\mathrm{dB},\;h_{sd}=h_{sr}=h_{rd}=h_{ds}=1,\;\sigma^{2}=\sigma_{r}^{2}=\sigma_{f}^{2}=1$). (**a**) Achievable rate $R_{\mathrm{GRC}}^{\mathrm{NFb}}\left(N,\epsilon \right)$ versus blocklength *N* and various decoding error probability ϵ ($P=10\;\mathrm{dB},P_{f}=20\;\mathrm{dB}$); (**b**) relationship between the achievable rate $R_{\mathrm{GRC}}^{\mathrm{NFb}}\left(N,\epsilon \right)$ and its asymptotic value $R_{\mathrm{GRC}}^{*}\left(N,\epsilon \right)$ ($P=10\;\mathrm{dB},\epsilon =10^{-6}$); (**c**) relationship between the relative rate ΔR and the relative power ΔP ($N=100,\epsilon =10^{-6}$).

Figure 4 shows the performance analysis of the SK-Type scheme for the GRC with noisy feedback. Here, the achievable rate $R_{\mathrm{GRC}}^{\mathrm{NFb}}\left(N,\epsilon \right)$ of this proposed scheme is given in (8). In Figure 4a, we show the relationship between the achievable rate $R_{\mathrm{GRC}}^{\mathrm{NFb}}\left(N,\epsilon \right)$, the coding blocklength *N* and the decoding error probability ϵ. From Figure 4a, we see that when the coding blocklength *N* and the decoding error probability ϵ increase, the achievable rate $R_{\mathrm{GRC}}^{\mathrm{NFb}}\left(N,\epsilon \right)$ also increases. Figure 4b depicts the relationship between the achievable rate $R_{\mathrm{GRC}}^{\mathrm{NFb}}\left(N,\epsilon \right)$ and its asymptotic value $R_{\mathrm{GRC}}^{*}\left(N,\epsilon \right)$. Figure 4b shows that as blocklength *N* increases, there exists an asymptotic value $R_{\mathrm{GRC}}^{*}\left(N,\epsilon \right)$ for the achievable rate $R_{\mathrm{GRC}}^{\mathrm{NFb}}\left(N,\epsilon \right)$. And as blocklength *N* and the power of the feedback channel $P_{f}$ increase, it will approach its asymptotic value more closely. This simulation result is the same as the theoretical result in Remark 1. Figure 4c illustrates the relationship between the relative rate ΔR and the relative power ΔP, where the relative rate ΔR and the relative power ΔP are, respectively, defined as

(68) $$\Delta R=\frac{R_{\mathrm{GRC}}^{\mathrm{NFb}}\left(N,\epsilon \right)}{R_{\mathrm{GRC}}^{\mathrm{PFb}}},\Delta P=\frac{P_{f}}{P}$$

where $R_{\mathrm{GRC}}^{\mathrm{NFb}}\left(N,\epsilon \right)$ is given in (8) and $R_{\mathrm{GRC}}^{\mathrm{PFb}}$ is given in (19). From Figure 4c, it shows that when the power of the Destination–Source feedback channel $P_{f}$ is sufficiently large, the achievable rate $R_{\mathrm{GRC}}^{\mathrm{NFb}}\left(N,\epsilon \right)$ of the noisy feedback scheme in Theorem 1 approaches the rate $R_{\mathrm{GRC}}^{\mathrm{PFb}}$ of the noiseless feedback scheme in [23].

**Figure 5:**
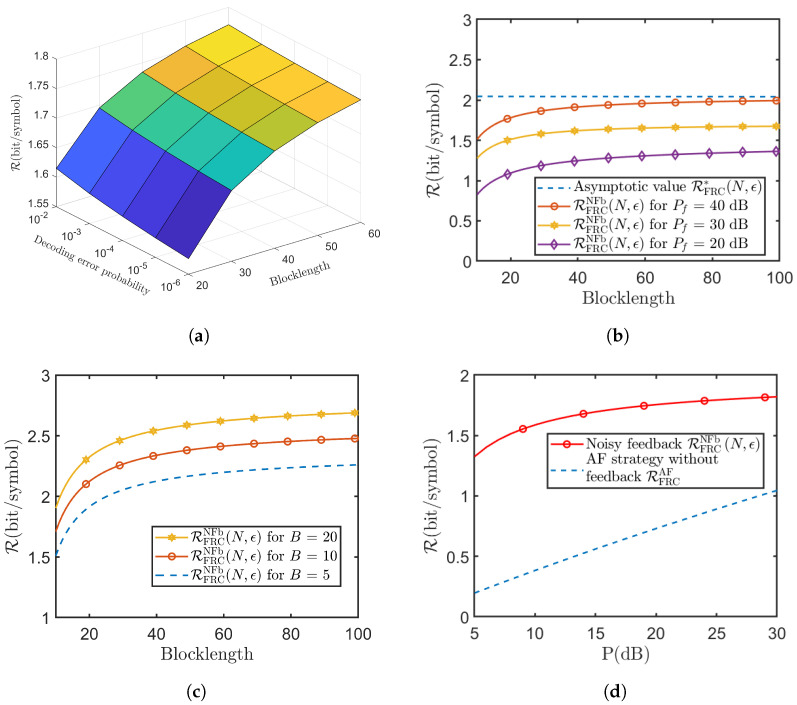
Performance analysis of the proposed scheme for the MISO FRC with noisy feedback ($P_{r}=10\;\mathrm{dB},\;h_{\mathrm{sd}},h_{\mathrm{sr}},h_{rd},h_{\mathrm{ds}}∼\mathrm{CN}\left(0,1\right),\;\sigma^{2}=\sigma_{r}^{2}=\sigma_{f}^{2}=1$). (**a**) Relationship among the achievable rate $R_{\mathrm{FRC}}^{\mathrm{NFb}}\left(N,\epsilon \right)$, blocklength *N* and decoding error probability ϵ ($B=5,P=10\;\mathrm{dB},P_{f}=20\;\mathrm{dB}$); (**b**) telationship between the achievable rate $R_{\mathrm{FRC}}^{\mathrm{NFb}}\left(N,\epsilon \right)$ and its asymptotic value $R_{\mathrm{FRC}}^{*}\left(N,\epsilon \right)$ ($P\;=\;10\;\mathrm{dB},\epsilon =10^{-6}$); (**c**) the influence of the number of Source’s antennas *B* on the achievable rate $R_{\mathrm{FRC}}^{\mathrm{NFb}}\left(N,\epsilon \right)$ ($P=10\;\mathrm{dB},P_{f}=20\;\mathrm{dB},\epsilon =10^{-6}$); (**d**) comparison of achievable rate for MISO FRC with noisy $R_{\mathrm{FRC}}^{\mathrm{NFb}}\left(N,\epsilon \right)$ and without feedback $R_{\mathrm{FRC}}^{\mathrm{AF}}$ ($N=100,\epsilon =10^{-6},B=5,P_{f}=20\;\mathrm{dB}$).

Figure 5 shows the performance analysis of the SK-Type scheme for the MISO FRC with noisy feedback. Here, the achievable rate $R_{\mathrm{FRC}}^{\mathrm{NFb}}\left(N,\epsilon \right)$ of this proposed scheme is given in (50). The conclusions drawn in Figure 4a,b are consistent with the results in the Gaussian case; i.e, when the coding blocklength *N* and the decoding error probability ϵ increase, the achievable rate $R_{\mathrm{FRC}}^{\mathrm{NFb}}\left(N,\epsilon \right)$ also increases. And as blocklength *N* increases, there exists an asymptotic value $R_{\mathrm{FRC}}^{*}\left(N,\epsilon \right)$ for the achievable rate $R_{\mathrm{FRC}}^{\mathrm{NFb}}\left(N,\epsilon \right)$. Figure 5c illustrates the influence of the number of the Source’s antennas *B* on the achievable rate $R_{\mathrm{FRC}}^{\mathrm{NFb}}\left(N,\epsilon \right)$ in the proposed SK-Type coding scheme. From Figure 5c, we conclude that as the number of the Source’s antennas *B* increases, the achievable rate $R_{\mathrm{FRC}}^{\mathrm{NFb}}\left(N,\epsilon \right)$ also increases, which indicates that the number of the Source’s antennas *B* can bring a gain to the achievable rate $R_{\mathrm{FRC}}^{\mathrm{NFb}}\left(N,\epsilon \right)$ of our proposed SK-Type coding scheme. Figure 5d compares the achievable rates of the SK-Type coding scheme for the MISO FRC with noisy feedback and the scheme without feedback [32], where both Relays use the AF strategy. Figure 5d shows that our proposed scheme can significantly enhance the achievable rate even in the presence of noise in the feedback channel.

**Figure 6:**
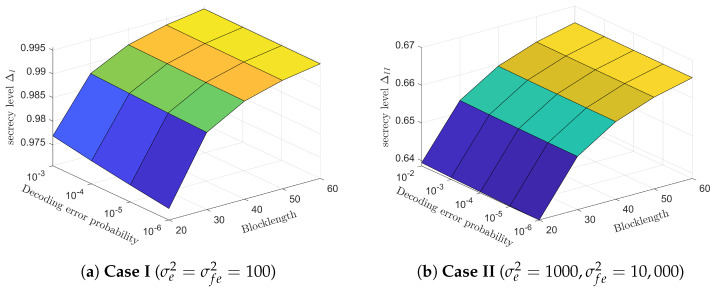
The relationship among the secrecy levels Δ, blocklength *N*, and decoding error probability ϵ of the proposed scheme for the MISO FRC with noisy feedback and a multi-antenna external Eavesdropper ($B=D=5,P=P_{r}=10\;\mathrm{dB},P_{f}=20\;\mathrm{dB},h_{\mathrm{sd}},h_{\mathrm{sr}},h_{rd},h_{\mathrm{ds}},g_{\mathrm{se}},g_{\mathrm{re}},g_{\mathrm{de}}∼\mathrm{CN}\left(0,1\right),\sigma^{2}=\sigma_{r}^{2}=\sigma_{f}^{2}=1$).

Figure 6 shows the relationship between secrecy level Δ, blocklength *N* and decoding error probability ϵ of the proposed SK-Type FBL coding scheme for the MISO FRC with noisy feedback and in the presence of Eavesdropper. From Figure 6a,b, we see that both the secrecy level in Case I $\Delta_{I}$ and Case II $\Delta_{II}$ increase with the increase of the blocklength *N* and decoding error probability $P_{e}$. To be specific, when the blocklength N=60 and decoding error probability $\epsilon =10^{-3}$, the secrecy level in case I $\Delta_{I}$ has already exceeded 0.99 (secrecy level equal to 1 corresponds to perfect secrecy). However, under the same parameters, the secrecy level in case II $\Delta_{II}$ only approaches 0.7. This result indicates that the maximum secrecy level achieved in case I (Eavesdropper does not know the complex dither signal sequence $V_{1}^{N-1}$) is superior to that of case II (Eavesdropper knows complex dither signal sequence $V_{1}^{N-1}$), which is consistent with the theoretical analysis.

**Figure 7:**
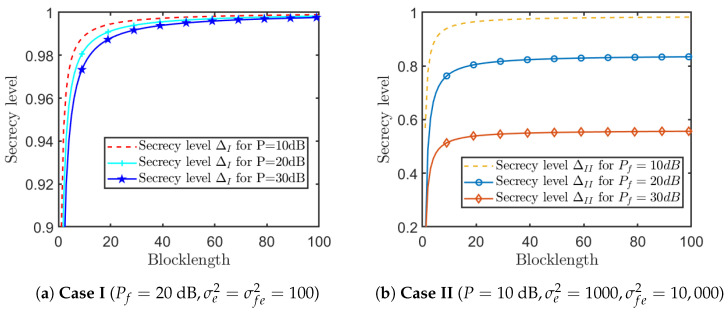
Relationship between the secrecy levels Δ and blocklength *N* under various power ($B=D=5,P_{r}=10\;\mathrm{dB},h_{\mathrm{sd}},h_{\mathrm{sr}},h_{rd},h_{\mathrm{ds}},g_{\mathrm{se}},g_{\mathrm{re}},g_{\mathrm{de}}∼\mathrm{CN}\left(0,1\right),\sigma^{2}=\sigma_{r}^{2}=\sigma_{f}^{2}=1,\epsilon =10^{-6}$).

Figure 7 shows the relationship between the secrecy level Δ and blocklength *N* under various powers of the proposed SK-Type FBL coding scheme. Figure 7a compares the secrecy level in Case I $\Delta_{I}$ under different powers of Source encoder *P*. It is easy to see that secrecy level in Case I $\Delta_{I}$ approaches perfect secrecy when the blocklength *N* is about 100, and a larger *P* causes secrecy performance degradation on small *N*. Figure 7b, plots secrecy level in Case II $\Delta_{II}$ under several powers of the feedback channel $P_{f}$. From this figure, we can see that the secrecy level in Case II $\Delta_{II}$ of the proposed scheme cannot achieve perfect secrecy in general cases. Actually, only when the channel quality of Destination-Eavesdropper is much poorer (i.e., $\sigma_{fe}^{2}\gg P_{f}$), the secrecy level in Case II $\Delta_{II}$ may approach 1 as the blocklength *N* tends to infinity.

## 6. Conclusions and Future Work

In this paper, we first proposed an SK-Type FBL coding scheme for the GRC with noisy feedback by introducing the lattice-based strategy to reduce the impact of the feedback channel noise. Numerical results show that when the power of the feedback channel is sufficiently large, the achievable rate almost approaches the rate of the GRC with noiseless feedback, and the required blocklength to achieve a desired decoding error probability is significantly short. Then, based on this scheme, we extended it to the MISO FRC with noisy feedback. The essence of this proposed scheme is to further extend the original real number scheme to the complex number. Simulation results show that even if there is noise in the feedback channel, this proposed scheme can still significantly improve the achievable rate of the MISO FRC without feedback. Finally, for the above MISO FRC with the noisy feedback model, we further consider multi-antenna Eavesdropper and derive a lower bound of the secrecy level in different cases by calculating the equivocation rate when Eavesdropper knows the complex dither signal sequence or not. We show that though perfect secrecy cannot be achieved in the noisy feedback model, it almost approaches perfect secrecy for some special cases. One possible future work is to extend this scheme to the fading MIMO case.

## Figures and Tables

**Figure A1:**
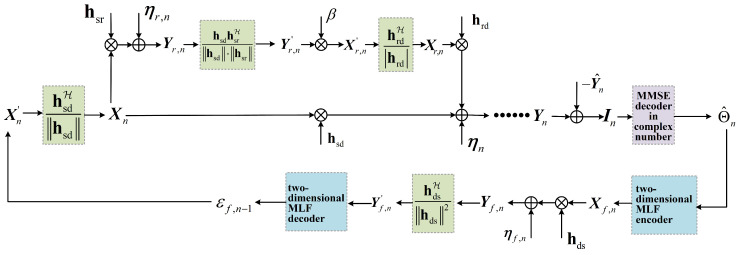
The schematic diagram of the proposed SK-type FBL scheme for the MISO GRC with noisy feedback.

## Data Availability

The data presented in this study are available on request from the corresponding author due to (specify the reason for the restriction).

## Appendix A. Coefficient’s Derivation and Error Probability Analysis of Theorem 1

Let $\hat{M}$ be the estimate of the transmitted message *M*, the decoder declares an error if $\hat{M}\neq M$. To determine the parameters, we need to analyze the error event in $P_{e}$. And the error events of the SK-Type FBL approach for the GRC with noisy feedback are described in Section 3.2.3, consisting of

(1) A modulo-aliasing error occurs in Source at time instant n(1≤n≤N−1). The event is defined as
  (A1) $$C_{n}≜\left\{\gamma_{n}\varepsilon_{n}+\frac{\eta_{f,n}}{h_{\mathrm{ds}}}\notin \left[-\frac{d}{2},\frac{d}{2}\right)\right\}.$$
(2) A decoding error occurs in Destination at time instant *N*. The event is defined as
  (A2) $$C_{N}≜\left\{\varepsilon_{N}\notin \left[-\frac{0.5}{2^{NR\left(N,\epsilon \right)}},\frac{0.5}{2^{NR\left(N,\epsilon \right)}}\right)\right\}.$$

Hence, by applying the union bound, we can derive

(A3) $$\begin{array}{cc} P_{e} & \leq \mathrm{Pr}\left(\overset{N}{\underset{n=1}{⋃}}C_{n}\right)\\ \; & =\mathrm{Pr}\left(\overset{N-1}{\underset{n=1}{⋃}}C_{n}\right)+\mathrm{Pr}\left(\overset{N-1}{\underset{n=1}{⋂}}{\bar{C}}_{n}\cap C_{N}\;\;\right)\\ \; & =\sum_{n=1}^{N-1}\mathrm{Pr}\left(\overset{n-1}{\underset{i=1}{⋂}}{\bar{C}}_{i}\cap C_{n}\right)+\mathrm{Pr}\left(\overset{N-1}{\underset{n=1}{⋂}}{\bar{C}}_{n}\cap C_{N}\right)\\ \; & ≜\sum_{n=1}^{N-1}\mathrm{Pr}\left({\tilde{C}}_{n}\right)+\mathrm{Pr}\left({\tilde{C}}_{N}\right), \end{array}$$

where $\bar{C}$ denotes the complement of the set *C*, ${\tilde{C}}_{n}≜{⋂}_{i=1}^{n-1}{\bar{C}}_{i}\cap C_{n}$, ${\tilde{C}}_{N}={⋂}_{n=1}^{N-1}{\bar{C}}_{n}\cap C_{N}$. $\mathrm{Pr}\left({\tilde{C}}_{n}\right)\;\left(1\leq n\leq N-1\right)$ represents the error probability that a modulo-aliasing occurs in Source at time instant *n* when there is no error occurring in all previous times, and $\mathrm{Pr}({\tilde{C}}_{N})$ represents the error probability of the final decoding without any modulo-aliasing error occurring in Source before time instance *N*.

For a given decoding error probability ϵ, let

(A4) $$\mathrm{Pr}\left({\tilde{C}}_{N}\right)=\frac{\epsilon }{2},\mathrm{Pr}\left({\tilde{C}}_{n}\right)=\frac{\epsilon }{2\left(N-1\right)}.$$

For the event ${\tilde{C}}_{n}\;\left(1\leq n\leq N-1\right)$, since no modulo-aliasing error occurs before time instant *n*, from (A1) and the fact that $\eta_{f,n}$ is Gaussian-distributed, we conclude that $\gamma_{n}\varepsilon_{n}+\eta_{f,n}/h_{\mathrm{ds}}∼N\left(0,\gamma_{n}^{2}\alpha_{n}+\sigma_{f}^{2}/h_{\mathrm{ds}}^{2}\right)$. From (A1), (A4) and $d=\sqrt{12P_{f}}$, we have

(A5) $$\begin{array}{cc} \mathrm{Pr}\left({\tilde{C}}_{n}\right) & =\mathrm{Pr}\left\{\gamma_{n}\varepsilon_{n}+\frac{\eta_{f,n}}{h_{\mathrm{ds}}}\notin \left[-\frac{d}{2},\frac{d}{2}\right)\right\}\\ \; & =2Q\left(\sqrt{\frac{3P_{f}}{\gamma_{n}^{2}\alpha_{n}+\frac{\sigma_{f}^{2}}{h_{ds}^{2}}}}\right). \end{array}$$

To simplify the calculation, we introduce a looseness parameter $L_{↑}$, which is denoted by

(A6) $$L_{↑}≜\frac{P_{f}}{\gamma_{n}^{2}\alpha_{n}+\frac{\sigma_{f}^{2}}{h_{ds}^{2}}}=\frac{1}{3}{\left(Q^{-1}\left(\frac{\epsilon }{4\left(N-1\right)}\right)\right)}^{2}.$$

Substituting (A4) and (A6) into (A5), we determine the appropriate selection of coefficient $\gamma_{n}\;\left(1\leq n\leq N-1\right)$,

(A7) $$\gamma_{n}=\sqrt{\left(\frac{P_{f}}{L_{↑}}-\frac{\sigma_{f}^{2}}{h_{ds}^{2}}\right)\frac{1}{\alpha_{n}}}.$$

According to (38), we obtain

(A8) $$\alpha_{f,n}≜\mathrm{Var}\left(\varepsilon_{f,n}\right)\overset{\left(a\right)}{=}\alpha_{n}+\frac{\sigma_{f}^{2}}{\gamma_{n}^{2}h_{ds}^{2}}\overset{\left(b\right)}{=}\alpha_{n}\frac{P_{f}h_{ds}^{2}}{P_{f}h_{ds}^{2}-L_{↑}\sigma_{f}^{2}}\overset{\left(c\right)}{=}\alpha_{n}\phi ,$$

where (a) follows from the fact that $\eta_{f,n}$ is dependent on $\varepsilon_{n}$, (b) follows from (A7), and (c) follows from $\phi ≜\frac{P_{f}h_{ds}^{2}}{P_{f}h_{ds}^{2}-L_{↑}\sigma_{f}^{2}}$.

To derive the achievable rate of this SK-type scheme, we focus on the final estimation error. At time instant 1, the estimation error and its variance are given in (25) and (26). So we consider the second moment and the following moments.

At time instant 2, we first need to calculate the MMSE estimation coefficient of Destination based on $Y_{2}$, that is,

(A9) $$\begin{array}{cc} \; & E\left[\varepsilon_{1}Y_{2}\right]=h_{sd}\sqrt{\frac{P}{\phi }}\sqrt{\alpha_{1}},\\ \; & E\left[Y_{2}^{2}\right]=h_{sd}^{2}\frac{P}{\phi }+h_{sd}^{2}P\left(1-\frac{1}{\phi }\right)+\sigma^{2}, \end{array}$$

and the estimation error is given in (34). So, the variance of $\varepsilon_{2}$ is

(A10) $$\begin{array}{cc} \alpha_{2} & ≜\mathrm{Var}\left(\varepsilon_{2}\right)=\alpha_{1}-\frac{{\left(E\left[\varepsilon_{1}Y_{2}\right]\right)}^{2}}{E\left[Y_{2}^{2}\right]}\\ \; & =\alpha_{1}{\left(1+\frac{h_{sd}^{2}\frac{P}{\phi }}{h_{sd}^{2}P\left(1-\frac{1}{\phi }\right)+\sigma^{2}}\right)}^{-1}, \end{array}$$

where $\alpha_{1}$ is given in (26).

At time instant n(3≤n≤N), similar to the second moment, we also calculate the MMSE estimation coefficient of Destination based on $I_{n}$ instead of $Y_{n}$; that is,

(A11) $$\begin{array}{cc} \; & E\left[\varepsilon_{n-1}I_{n}\right]=\sqrt{\alpha_{n-1}}\left(h_{sd}\sqrt{\frac{P}{\phi }}+h_{sr}h_{rd}\beta_{↑}\sqrt{P}\rho_{↑n-1}\right),\\ \; & E\left[I_{n}^{2}\right]={\left(h_{sd}\sqrt{\frac{P}{\phi }}+h_{sr}h_{rd}\beta_{↑}\sqrt{P}\rho_{↑n-1}\right)}^{2}\\ \; & +h_{sr}^{2}h_{rd}^{2}\beta_{↑}^{2}P\left(\left(1-\frac{1}{\phi }\right)-{\left(1-\frac{1}{A_{↑n-1}}\right)}^{2}\rho_{↑n-1}^{2}\right)+F_{↑}, \end{array}$$

where $F_{↑}=h_{sd}^{2}P\left(1-\frac{1}{\phi }\right)+h_{rd}^{2}\beta_{↑}^{2}\sigma_{r}^{2}+\sigma^{2}$.

The estimation error at time instant n(3≤n≤N) is given in (46), so the variance of $\varepsilon_{n}$ is

(A12) $$\begin{array}{cc} \; & \alpha_{n}≜\mathrm{Var}\left(\varepsilon_{n}\right)=\alpha_{n-1}-\frac{{\left(E\left[\varepsilon_{n-1}I_{n}\right]\right)}^{2}}{E\left[I_{n}^{2}\right]}=\alpha_{n-1}\times \\ \; & {\left(1+\frac{{\left(h_{sd}\sqrt{\frac{P}{\phi }}+h_{sr}h_{rd}\beta_{↑}\sqrt{P}\rho_{↑n-1}\right)}^{2}}{h_{sr}^{2}h_{rd}^{2}\beta_{↑}^{2}P\left(\left(1-\frac{1}{\phi }\right)-{\left(1-\frac{1}{A_{↑n-1}}\right)}^{2}\rho_{↑n-1}^{2}\right)+F_{↑}}\right)}^{-1}, \end{array}$$

where $\rho_{↑n-1}$ is the correlation coefficient between $\varepsilon_{n-1}$ and $\varepsilon_{f,n-2}$, denoted by

(A13) $$\rho_{↑n-1}≜\frac{E\left[\varepsilon_{n-1}\varepsilon_{f,n-2}\right]}{\sqrt{\alpha_{n-1}}\sqrt{\alpha_{f,n-2}}}=A_{↑n-1}\sqrt{\frac{\alpha_{n-1}}{\alpha_{f,n-2}}},$$

and

(A14) $$A_{↑n-1}=1+\frac{Ph_{sd}\left(h_{sd}\sqrt{\frac{1}{\phi }}+h_{sr}h_{rd}\beta_{↑}\rho_{↑n-2}\right)\left(\sqrt{\frac{1}{\phi }}-\sqrt{\phi }\right)}{h_{sr}^{2}h_{rd}^{2}\beta_{↑}^{2}P\left(\left(1-\frac{1}{\phi }\right)-{\left(1-\frac{1}{A_{↑n-2}}\right)}^{2}\rho_{↑n-2}^{2}\right)+F_{↑}}.$$

It is worth noting that the initial value of $\rho_{↑n-1}$ is given by

(A15) $$\rho_{↑2}=\frac{\sigma^{2}}{h_{sd}^{2}P\left(1-\frac{1}{\phi }\right)+\sigma^{2}}\sqrt{\frac{\alpha_{2}}{\alpha_{1}\phi }}.$$

From (A13) and (A14), it is apparent that $\left|\rho_{↑n}\right|\neq \left|\rho_{↑n-1}\right|$. By applying **Cesáro’s Mean Theorem** in [20] to the $\left\{\rho_{↑n}\right\}$, it follows that this sequence will converge to a unique fixed point in $\left[0,1\right]$. Hence, we obtain the following equation

(A16) $$\begin{array}{cc} \; & \rho_{↑}^{2}=\frac{{\left(A_{↑}\left(\rho_{↑}\right)\right)}^{2}}{\phi }\times \\ \; & {\left(1+\frac{{\left(h_{sd}\sqrt{\frac{P}{\phi }}+h_{sr}h_{rd}\beta_{↑}\sqrt{P}\rho_{↑}\right)}^{2}}{h_{sr}^{2}h_{rd}^{2}\beta_{↑}^{2}P\left(\left(1-\frac{1}{\phi }\right)-{\left(1-\frac{1}{A_{↑}\left(\rho_{↑}\right)}\right)}^{2}\rho_{↑}^{2}\right)+F_{↑}}\right)}^{-1}, \end{array}$$

where

(A17) $$A_{↑}\left(\rho_{↑}\right)=1+\frac{Ph_{sd}\left(h_{sd}\sqrt{\frac{1}{\phi }}+h_{sr}h_{rd}\beta_{↑}\rho_{↑}\right)\left(\sqrt{\frac{1}{\phi }}-\sqrt{\phi }\right)}{h_{sr}^{2}h_{rd}^{2}\beta_{↑}^{2}P\left(\left(1-\frac{1}{\phi }\right)-{\left(1-\frac{1}{A_{↑}\left(\rho_{↑}\right)}\right)}^{2}\rho_{↑}^{2}\right)+F_{↑}}.$$

Let $\rho_{↑}^{*}$ be defined as the unique solution within $\left[0,1\right]$ of (A16). Then, according to (A12), the variance of the final estimation error $\varepsilon_{N}$ is as follows

(A18) $$\alpha_{N}=\alpha_{2}{\left(1+\frac{{\left(h_{sd}\sqrt{\frac{P}{\phi }}+h_{sr}h_{rd}\beta_{↑}\sqrt{P}\rho_{↑}^{*}\right)}^{2}}{\Gamma_{↑}}\right)}^{2-N},$$

where

(A19) $$\Gamma_{↑}=h_{sr}^{2}h_{rd}^{2}\beta_{↑}^{2}P\left(\left(1-\frac{1}{\phi }\right)-{\left(1-\frac{1}{A_{↑}\left(\rho_{↑}^{*}\right)}\right)}^{2}{\left(\rho_{↑}^{*}\right)}^{2}\right)+F_{↑}.$$

Next, according to (A2) and (A4), we have

(A20) $$\begin{array}{cc} \mathrm{Pr}\left({\tilde{C}}_{N}\right) & =\mathrm{Pr}\left\{\varepsilon_{N}\notin \left[-\frac{0.5}{2^{NR\left(N,\epsilon \right)}},\frac{0.5}{2^{NR\left(N,\epsilon \right)}}\right)\right\}\\ \; & =2Q\left(\frac{0.5}{2^{NR\left(N,\epsilon \right)}\sqrt{\alpha_{N}}}\right)=\frac{\epsilon }{2}. \end{array}$$

Substituting (A18) and (A19) into (A20), we obtain

(A21) $$R_{\mathrm{GRC}}^{\mathrm{NFb}}\left(N,\epsilon \right)=R_{\mathrm{GRC}}^{*}\left(N,\epsilon \right)-R_{\mathrm{GRC}}^{◊}\left(N,\epsilon \right),$$

where $R_{\mathrm{GRC}}^{*}\left(N,\epsilon \right)$ and $R_{\mathrm{GRC}}^{◊}\left(N,\epsilon \right)$ are, respectively, given in (9) and (10). It should be noted that $R_{\mathrm{GRC}}^{*}\left(N,\epsilon \right)$ represents the maximum achievable rate when blocklength *N* is sufficiently large. And $R_{\mathrm{GRC}}^{◊}\left(N,\epsilon \right)$ represents the achievable rate loss caused by given blocklength *N* and decoding error probability ϵ.

## Appendix B. Proof of Theorem 2

### Appendix B.1. Channel Transformation Strategy

**Pre-coding strategy for the feedforward MISO channels:** At time instant n(1≤n≤N), the output signal received by Destination is given in (6). Let

(A22) $$\begin{array}{cc} X_{n}=\frac{h_{\mathrm{sd}}^{H}}{∥h_{\mathrm{sd}}∥}X_{n}^{{}^{\prime }} & ⟹X_{n}^{{}^{\prime }}=\frac{h_{\mathrm{sd}}}{∥h_{\mathrm{sd}}∥}X_{n},\\ X_{r,n}=\frac{{h_{rd}}^{H}}{\left|h_{rd}\right|}{X_{r,n}}^{{}^{\prime }} & ⟹{X_{r,n}}^{{}^{\prime }}=\frac{h_{rd}}{\left|h_{rd}\right|}X_{r,n}, \end{array}$$

where $h_{\mathrm{sd}}\in C^{1\times B},h_{rd}\in C^{1\times 1},X_{n}^{{}^{\prime }}\in C^{1\times 1},{X_{r,n}}^{{}^{\prime }}\in C^{1\times 1}$, and

(A23) $$\begin{array}{cc} E\left[X_{n}^{H}X_{n}\right] & =E\left[\frac{X_{n}^{{}^{\prime }H}h_{\mathrm{sd}}}{∥h_{\mathrm{sd}}∥}\cdot \frac{h_{\mathrm{sd}}^{H}X_{n}^{{}^{\prime }}}{∥h_{\mathrm{sd}}∥}\right]=E\left[|X_{n}^{{}^{\prime }}{|}^{2}\right]=P,\\ E\left[{X_{r,n}}^{{}^{\prime }}{X_{r,n}}^{{}^{\prime }H}\right] & =E\left[\frac{h_{rd}X_{r,n}}{\left|h_{rd}\right|}\cdot \frac{X_{r,n}^{H}h_{\mathrm{rd}}^{H}}{\left|h_{rd}\right|}\right]=E\left[X_{r,n}X_{r,n}^{H}\right]=P_{r}, \end{array}$$

which indicates that the power constraint of $X_{n}^{{}^{\prime }}$ and ${X_{r,n}}^{{}^{\prime }}$ are exactly the same as that of $X_{n},X_{r,n}$, respectively. Therefore, by substituting (A22) into (6), the signal received by Destination can be further expressed as

(A24) $$\begin{array}{cc} Y_{n} & =h_{\mathrm{sd}}X_{n}+h_{rd}X_{r,n}+\eta_{n},\\ \Rightarrow Y_{n} & =h_{\mathrm{sd}}\frac{h_{\mathrm{sd}}^{H}}{∥h_{\mathrm{sd}}∥}X_{n}^{{}^{\prime }}+h_{rd}\frac{{h_{rd}}^{H}}{\left|h_{rd}\right|}{X_{r,n}}^{{}^{\prime }}+\eta_{n},\\ \Rightarrow Y_{n} & =∥h_{\mathrm{sd}}∥X_{n}^{{}^{\prime }}+\left|h_{rd}\right|{X_{r,n}}^{{}^{\prime }}+\eta_{n}. \end{array}$$

Specifically, the link from Source to Relay is also a MISO channel, and the messages sent by Source and Relay are related. So, at time instant $n\;\left(1\leq n\leq N\right)$, the signal received by Relay can be further expressed as

(A25) $$\begin{array}{cc} \; & Y_{r,n}=h_{\mathrm{sr}}X_{n}+\eta_{r,n}=h_{\mathrm{sr}}\frac{h_{\mathrm{sd}}^{H}}{∥h_{\mathrm{sd}}∥}X_{n}^{{}^{\prime }}+\eta_{r,n},\\ \Rightarrow & \frac{h_{\mathrm{sd}}h_{\mathrm{sr}}^{H}}{∥h_{\mathrm{sd}}∥\cdot ∥h_{\mathrm{sr}}∥}Y_{r,n}=∥h_{\mathrm{sr}}∥X_{n}^{{}^{\prime }}+\frac{h_{\mathrm{sd}}h_{\mathrm{sr}}^{H}}{∥h_{\mathrm{sd}}∥\cdot ∥h_{\mathrm{sr}}∥}\eta_{r,n},\\ \Rightarrow & {Y_{r,n}}^{{}^{\prime }}=∥h_{\mathrm{sr}}∥X_{n}^{{}^{\prime }}+\eta_{r,n}^{{}^{\prime }}, \end{array}$$

where $h_{\mathrm{sr}}\in C^{1\times B},h_{\mathrm{sd}}\in C^{1\times B},X_{n}^{{}^{\prime }}\in C^{1\times 1}$, and

(A26) $$Y_{r,n}^{{}^{\prime }}=\frac{h_{\mathrm{sd}}h_{\mathrm{sr}}^{H}}{∥h_{\mathrm{sd}}∥\cdot ∥h_{\mathrm{sr}}∥}Y_{r,n},\;\;\;\eta_{r,n}^{{}^{\prime }}=\frac{h_{\mathrm{sd}}h_{\mathrm{sr}}^{H}}{∥h_{\mathrm{sd}}∥\cdot ∥h_{\mathrm{sr}}∥}\eta_{r,n},$$

$Y_{r,n}^{{}^{\prime }}\in C^{1\times 1},\eta_{r,n}^{{}^{\prime }}\in C^{1\times 1}∼\mathrm{CN}\left(0,\sigma_{r}^{2}\right)$. It indicates that the complex-valued feedforward MISO FRC can be transformed into a complex-valued single-input single-output (SISO) FRC case.

**Beamforming strategy for the feedback SIMO channel:** At time $n\;\left(1\leq n\leq N-1\right)$, the signal received by Source (see (6)) can be proceeded as

(A27) $$\begin{array}{cc} \; & Y_{f,n}=h_{\mathrm{ds}}X_{f,n}+\eta_{f,n},\\ \Rightarrow & \frac{h_{\mathrm{ds}}^{H}}{{∥h_{\mathrm{ds}}∥}^{2}}Y_{f,n}=X_{f,n}+\frac{h_{\mathrm{ds}}^{H}}{{∥h_{\mathrm{ds}}∥}^{2}}\eta_{f,n},\\ \Rightarrow & Y_{f,n}^{{}^{\prime }}=X_{f,n}+\eta_{f,n}^{{}^{\prime }}, \end{array}$$

where $h_{\mathrm{ds}}\in C^{B\times 1},X_{f,n}\in C^{1\times 1},Y_{f,n}^{{}^{\prime }}=\frac{h_{\mathrm{ds}}^{H}Y_{f,n}}{{∥h_{\mathrm{ds}}∥}^{2}}\in C^{1\times 1},\eta_{f,n}^{{}^{\prime }}=\frac{h_{\mathrm{ds}}^{H}\eta_{f,n}}{{∥h_{\mathrm{ds}}∥}^{2}}\in C^{1\times 1}∼\mathrm{CN}\left(0,\frac{\sigma_{f}^{2}}{{∥h_{\mathrm{ds}}∥}^{2}}\right)$. This indicates that the complex-valued feedback SIMO channel can be transformed into complex-valued SISO channels.

Therefore, according to (A24), (A25) and (A27), we complete the transformation of MISO FRC with noisy feedback into the SISO FRC with noisy feedback.

### Appendix B.2. Message Mapping Method

In the proposed SK-Type FBL scheme for the MISO FRC with noisy feedback, we extend the one-dimensional real number axis mapping in the GRC with noisy feedback to the two-dimensional complex plane mapping. To be specific, for given coding blocklength *N* and decoding error probability ϵ, let $\left|M\right|=2^{NR(N,\epsilon )}$, where *M* is the transmitted message uniformly distributed over the set $M=\{1,2,\ldots ,2^{NR(N,\epsilon )}\}$. We divide a complex plane region [±0.5,±0.5j] into $2^{NR\left(N,\epsilon \right)}$ equally spaced sub-complex plane regions with both the length and width of each sub-complex plane region being $2^{-NR(N,\epsilon )/2}$. The message *M* is one-to-one mapped to the center point of each sub-complex plane region, denoted as $\Theta =\theta_{R}+j\theta_{I}$, where $\theta_{R}$ and $\theta_{I}$, respectively, represent the real and imaginary parts of the complex element Θ, *j* is the imaginary unit. Since *M* is equi-probably distributed over the set M, $\theta_{R}$ and $\theta_{I}$ are both approximately uniformly distributed over the interval [−0.5,0.5] (i.e., $\theta_{R},\theta_{I}∼\mathrm{Unif}\left[-0.5,0.5\right]$), and their variances approximately equal 1/12, i.e., $E\left[\theta_{R}^{2}\right]=E\left[\theta_{I}^{2}\right]=1/12$, so

(A28) $$\mathrm{Var}\left(\Theta \right)=E\left[\Theta \Theta^{H}\right]=E\left[\theta_{R}^{2}\right]+E\left[\theta_{I}^{2}\right]=\frac{1}{6}.$$

### Appendix B.3. Dither Signal for the Feedback Channel Power Constaint

In this proposed scheme, we further extend the dither signal in the real number (see in Section 3.2.2) to the complex dither signal $V_{n}\;\left(1\leq n\leq N-1\right)$ to ensure the codewords encoded by Destination satisfy the power constraints at time *n*. It is also perfectly known by Source and Destination, and it is i.i.d. generated and uniformly distributed over Λ, where $\Lambda =\Lambda_{R}+j\Lambda_{I}$ with Λ∈[±d/2,±jd/2). Due to the average power constraint of $X_{n}\;\left(1\leq n\leq N-1\right)$ on the feedback channel encoder, the value of *d* is $\sqrt{6P_{f}}$. In addition, $\gamma_{n}\;\left(1\leq n\leq N-1\right)$ is the real-valued number and is chosen to avoid modulo-aliasing errors occurring in Source.

### Appendix B.4. Coding Procedure

The specific coding process is similar to what was described in Section 3.2.3. For the similar parts, we omit them directly, focusing here on elaborating the distinctions from Section 3.2.3.

*After channel transformation, at time *1**,

- Source sends $X_{1}^{{}^{\prime }}=\sqrt{6P}\Theta$, the relay keeps quiet and Destination receives $Y_{1}=∥h_{\mathrm{sd}}∥X_{1}^{{}^{\prime }}+\eta_{1}=∥h_{\mathrm{sd}}∥\sqrt{6P}\Theta +\eta_{1}$.
- Once it receives $Y_{1}$, Destination first computes the estimation of Θ by ${\hat{\Theta }}_{1}=\frac{Y_{1}}{∥h_{\mathrm{sd}}∥\sqrt{6P}}=\Theta +\frac{\eta_{1}}{∥h_{\mathrm{sd}}∥\sqrt{6P}}≜\Theta +\varepsilon_{1}$. The first estimation error is denoted by $\varepsilon_{1}≜{\hat{\Theta }}_{1}-\Theta =\frac{\eta_{1}}{∥h_{\mathrm{sd}}∥\sqrt{6P}}$, and the variance of $\varepsilon_{1}$ is $\alpha_{1}≜\mathrm{Var}\left(\varepsilon_{1}\right)=\frac{\sigma^{2}}{6P{∥h_{\mathrm{sd}}∥}^{2}}$.
- Then, Destination uses the two-dimensional *modulo*-Λ function to encode ${\hat{\Theta }}_{1}$ as $X_{f,1}=M_{\Lambda }\left[\gamma_{1}{\hat{\Theta }}_{1}+V_{1}\right]$ and transmits it back to Source via a noisy feedback channel.

*At time *2**,

- Once receiving $Y_{f,1}^{{}^{\prime }}$, Source also uses the two-dimensional *modulo*-Λ function to decode this message and computes the noisy versions of Destination’s estimation error, i.e., $\varepsilon_{f,1}=\frac{1}{\gamma_{1}}M_{\Lambda }\left[Y_{f,1}^{{}^{\prime }}-\gamma_{1}\Theta -V_{1}\right]=\frac{1}{\gamma_{1}}M_{\Lambda }\left[\gamma_{1}\varepsilon_{1}+\eta_{f,1}^{{}^{\prime }}\right]$. If the modulo-aliasing error does not occur, using Property (2) of Proposition 2, Source can obtain $\varepsilon_{f,1}=\varepsilon_{1}+\eta_{f,1}^{{}^{\prime }}/\gamma_{1}$.
- The Source sends $X_{2}^{{}^{\prime }}=\sqrt{P/\alpha_{f,1}}\varepsilon_{f,1}$ to Destination, where $\alpha_{f,1}≜\mathrm{Var}\left(\varepsilon_{f,1}\right)$. At this time instant, Relay also keeps quiet, so Destination receives the output signal $Y_{2}=∥h_{\mathrm{sd}}∥X_{2}^{{}^{\prime }}+\eta_{2}=∥h_{\mathrm{sd}}∥\sqrt{P/\alpha_{f,1}}\varepsilon_{f,1}+\eta_{2}$.
- Next, Destination evaluates MMSE of $\varepsilon_{1}$ based on $Y_{2}$ and updates the estimation of Θ by computing ${\hat{\Theta }}_{2}={\hat{\Theta }}_{1}-\frac{E\left[\varepsilon_{1}{Y_{2}}^{H}\right]}{E\left[Y_{2}{Y_{2}}^{H}\right]}Y_{2}=\Theta +\varepsilon_{2}$. The second estimation error is denoted by $\varepsilon_{2}≜{\hat{\Theta }}_{2}-\Theta =\varepsilon_{1}-\frac{E\left[\varepsilon_{1}{Y_{2}}^{H}\right]}{E\left[Y_{2}{Y_{2}}^{H}\right]}Y_{2}$, and the variance of $\varepsilon_{2}$ is $\alpha_{2}≜\mathrm{Var}\left(\varepsilon_{2}\right)$. Then, Destination encodes $X_{f,2}=M_{\Lambda }\left[\gamma_{2}{\hat{\Theta }}_{2}+V_{2}\right]$ and transmits it back to Source via a noisy feedback channel.

*At time n(3≤n≤N)*,

- Once it receives $Y_{f,n-1}^{{}^{\prime }}$, Source computes the noisy versions of Destination’s estimation error, i.e., $\varepsilon_{f,n-1}=\frac{1}{\gamma_{n-1}}M_{\Lambda }\left[\gamma_{n-1}\varepsilon_{n-1}+\eta_{f,n-1}^{{}^{\prime }}\right]$. If the modulo-aliasing error does not occur, using Property (2) of Proposition 2, Source can get $\varepsilon_{f,n-1}=\varepsilon_{n-1}+\eta_{f,n-1}^{{}^{\prime }}/\gamma_{n-1}$.
- The Source sends $X_{n}^{{}^{\prime }}=\sqrt{P/\alpha_{f,n-1}}\varepsilon_{f,n-1}$ to both the Relay and Destination, where $\alpha_{f,n-1}≜\mathrm{Var}\left(\varepsilon_{f,n-1}\right)$. Furthermore, Relay does not keep quiet and utilizes the AF strategy to transmit a scaled version of $Y_{r,n-1}^{{}^{\prime }}$; that is, ${X_{r,n}}^{{}^{\prime }}=\beta Y_{r,n-1}^{{}^{\prime }}=\beta \left(∥h_{\mathrm{sr}}∥X_{n-1}^{{}^{\prime }}+\eta_{r,n-1}^{{}^{\prime }}\right)=\beta \left(∥h_{\mathrm{sr}}∥\sqrt{P/\alpha_{f,n-2}}\varepsilon_{f,n-2}+\eta_{r,n-1}^{{}^{\prime }}\right)$, and the value of the Relay’s AF coefficient is $\beta =\sqrt{P_{r}/\left({∥h_{\mathrm{sr}}∥}^{2}P+\sigma_{r}^{2}\right)}$.
- Next, the *n*-th channel output signal of Destination received is $Y_{n}=∥h_{\mathrm{sd}}∥X_{n}^{{}^{\prime }}+\left|h_{rd}\right|{X_{r,n}}^{{}^{\prime }}+\eta_{n}=∥h_{\mathrm{sd}}∥\sqrt{P/\alpha_{f,n-1}}\varepsilon_{f,n-1}+\left|h_{rd}\right|\beta \left(∥h_{\mathrm{sr}}∥\sqrt{P/\alpha_{f,n-2}}\varepsilon_{f,n-2}+\eta_{r,n-1}^{{}^{\prime }}\right)+\eta_{n}$. Destination first calculates the auxiliary signal about the received message ${\hat{Y}}_{n}=∥h_{\mathrm{sr}}∥\cdot \left|h_{rd}\right|\beta \sqrt{P/\alpha_{f,n-2}}{\hat{\varepsilon }}_{n-2}$, then forms the innovation $I_{n}$ based on the fact that $\varepsilon_{n-1}=\varepsilon_{n-2}-{\hat{\varepsilon }}_{n-2}$. $I_{n}$ is denoted by $I_{n}≜Y_{n}-{\hat{Y}}_{n}=∥h_{\mathrm{sd}}∥\sqrt{P/\alpha_{f,n-1}}\varepsilon_{f,n-1}+\left|h_{rd}\right|\beta \left(∥h_{\mathrm{sr}}∥\sqrt{P/\alpha_{f,n-2}}\left(\varepsilon_{f,n-1}+\eta_{f,n-2}^{{}^{\prime }}/\gamma_{n-2}\right)+\eta_{r,n-1}^{{}^{\prime }}\right)+\eta_{n}$.
- Then Destination evaluates MMSE of $\varepsilon_{n-1}$ based on $I_{n}$ and updates the estimation of Θ by computing ${\hat{\Theta }}_{n}={\hat{\Theta }}_{n-1}-\frac{E\left[\varepsilon_{n-1}I_{n}\right]}{E\left[I_{n}^{2}\right]}I_{n}=\Theta +\varepsilon_{n}$. The estimation error at time instant n(3≤n≤N) is denoted by $\varepsilon_{n}≜{\hat{\Theta }}_{n}-\Theta =\varepsilon_{n-1}-\frac{E\left[\varepsilon_{n-1}I_{n}\right]}{E\left[I_{n}^{2}\right]}I_{2}$, and the variance of $\varepsilon_{n}$ is $\alpha_{n}≜\mathrm{Var}\left(\varepsilon_{n}\right)$.

Finally, at time instant *N*, Destination computes the final estimation ${\hat{\Theta }}_{N}=\Theta +\varepsilon_{N}$. According to the mapping method in Appendix B.2, if the final estimation error satisfies both of the following conditions

(A29) $$\begin{array}{c} \varepsilon_{R,N}\in \left[-\frac{0.5}{2^{\frac{NR\left(N,\epsilon \right)}{2}}},\frac{0.5}{2^{\frac{NR\left(N,\epsilon \right)}{2}}}\right),\;\;\;\varepsilon_{I,N}\in \left[-\frac{0.5}{2^{\frac{NR\left(N,\epsilon \right)}{2}}},\frac{0.5}{2^{\frac{NR\left(N,\epsilon \right)}{2}}}\right), \end{array}$$

the final estimation ${\hat{\Theta }}_{N}$ is closest to the Θ, and thus Destination successfully decodes the message *M*.

For a better understanding, the schematic diagram of the scheme proposed above is shown in Figure A1.

The coefficient’s derivation and the analysis of error probability for Theorem 2 are fundamentally consistent. We omit the similar parts directly and focus on elaborating the differences between them. See details below.

### Appendix B.5. Coefficient’s Derivation and Error Probability Analysis

The error events of the SK-type FBL coding scheme for the MISO FRC with noisy feedback described in Appendix B.4 consist of

(1) A modulo-aliasing error occurs in Source at time instant n(1≤n≤N−1). The event is defined as
  (A30) $$C_{n}≜\left\{\gamma_{n}\varepsilon_{n}+\eta_{f,n}^{{}^{\prime }}\notin \Lambda \right\},$$
  where $\varepsilon_{n}$ and $\eta_{f,n}^{{}^{\prime }}$ are both complex numbers, so (A30) can be further expressed as
  (A31) $$\begin{array}{cc} C_{n}≜ & \left\{\gamma_{n}\varepsilon_{R,n}+\eta_{fR,n}^{{}^{\prime }}\notin \left[-\frac{d}{2},\frac{d}{2}\right)\right\}⋃\\ \; & \left\{\gamma_{n}\varepsilon_{I,n}+\eta_{fI,n}^{{}^{\prime }}\notin \left[-\frac{d}{2},\frac{d}{2}\right)\right\}, \end{array}$$
  where $d=\sqrt{6P_{f}}$ and the subscripts *R* and *I* represent the real and imaginary parts of the original complex elements, respectively.
(2) A decoding error occurs in Destination at time instant *N*. The event is defined as
  (A32) $$\begin{array}{cc} C_{N}≜ & \left\{\varepsilon_{R,N}\notin \left[-\frac{0.5}{2^{\frac{NR\left(N,\epsilon \right)}{2}}},\frac{0.5}{2^{\frac{NR\left(N,\epsilon \right)}{2}}}\right)\right\}⋃\\ \; & \left\{\varepsilon_{I,N}\notin \left[-\frac{0.5}{2^{\frac{NR\left(N,\epsilon \right)}{2}}},\frac{0.5}{2^{\frac{NR\left(N,\epsilon \right)}{2}}}\right)\right\}. \end{array}$$

Hence, the error probability is bounded by (A3). And for a given decoding error probability ϵ, from (A30)–(A32), let

(A33) $$\mathrm{Pr}\left({\tilde{C}}_{N}\right)=\frac{\epsilon }{2},\mathrm{Pr}\left({\tilde{C}}_{n}\right)=\frac{\epsilon }{2\left(N-1\right)}.$$

Since no modulo-aliasing error occurs at time instant n(1≤n≤N−1), from (A30) and (A31) and the fact that $\eta_{f,n}^{{}^{\prime }}$ is a circularly symmetric Gaussian distribution, we conclude that $\gamma_{n}\varepsilon_{R,n}+\eta_{fR,n}^{{}^{\prime }}∼N\left(0,\left(\gamma_{n}^{2}\alpha_{n}+\sigma_{f}^{2}/{∥h_{\mathrm{ds}}∥}^{2}\right)/2\right)$, $\gamma_{n}\varepsilon_{I,n}+\eta_{fI,n}^{{}^{\prime }}∼N\left(0,\left(\gamma_{n}^{2}\alpha_{n}+\sigma_{f}^{2}/{∥h_{\mathrm{ds}}∥}^{2}\right)/2\right)$. So we have

(A34) $$\begin{array}{cc} \mathrm{Pr}\left({\tilde{C}}_{n}\right) & =2\mathrm{Pr}\left\{\gamma_{n}\varepsilon_{R,n}+\eta_{fR,n}^{{}^{\prime }}\notin \left[-\frac{d}{2},\frac{d}{2}\right)\right\}\\ \; & =4Q\left(\sqrt{\frac{3P_{f}}{\gamma_{n}^{2}\alpha_{n}+\frac{\sigma_{f}^{2}}{{∥h_{\mathrm{ds}}∥}^{2}}}}\right), \end{array}$$

where $d=\sqrt{6P_{f}}$.

Similar to the GRC scenario, we also introduce a looseness parameter *L*, which is denoted by

(A35) $$L≜\frac{P_{f}}{\gamma_{n}^{2}\alpha_{n}+\frac{\sigma_{f}^{2}}{{∥h_{\mathrm{ds}}∥}^{2}}}=\frac{1}{3}{\left(Q^{-1}\left(\frac{\epsilon }{8\left(N-1\right)}\right)\right)}^{2}.$$

Then, we determine the appropriate selection of coefficient $\gamma_{n}$,

(A36) $$\gamma_{n}=\sqrt{\left(\frac{P_{f}}{L}-\frac{\sigma_{f}^{2}}{{∥h_{\mathrm{ds}}∥}^{2}}\right)\frac{1}{\alpha_{n}}},$$

where $\gamma_{n}$ is the real-value number.

According to $\alpha_{f,n}≜\mathrm{Var}\left(\varepsilon_{f,n}\right)$, we obtain

(A37) $$\alpha_{f,n}=\alpha_{n}\frac{P_{f}{∥h_{\mathrm{ds}}∥}^{2}}{P_{f}{∥h_{\mathrm{ds}}∥}^{2}-L\sigma_{f}^{2}}\overset{\left(a\right)}{=}\alpha_{n}\Phi .$$

where (a) follows from $\Phi ≜P_{f}{∥h_{\mathrm{ds}}∥}^{2}/\left(P_{f}{∥h_{\mathrm{ds}}∥}^{2}-L\sigma_{f}^{2}\right)$.

The computation process of estimation errors at each time instant is analogous to that described in Appendix A. Here, we will omit the detailed procedures and provide the results directly.

At time instant 1, $\varepsilon_{1}=\frac{\eta_{1}}{\sqrt{6P}∥h_{\mathrm{sd}}∥}$ and $\alpha_{1}=\frac{\sigma^{2}}{6P{∥h_{\mathrm{sd}}∥}^{2}}$.

At time instant 2, $\varepsilon_{2}=\varepsilon_{1}-\frac{E[\varepsilon_{1}{Y_{2}}^{H}]}{E[Y_{2}{Y_{2}}^{H}]}Y_{2}$, where

(A38) $$\begin{array}{cc} \; & E\left[\varepsilon_{1}{Y_{2}}^{H}\right]=∥h_{\mathrm{sd}}∥\sqrt{\frac{P}{\Phi }}\sqrt{\alpha_{1}},\\ \; & E\left[Y_{2}{Y_{2}}^{H}\right]={∥h_{\mathrm{sd}}∥}^{2}\frac{P}{\Phi }+{∥h_{\mathrm{sd}}∥}^{2}P\left(1-\frac{1}{\Phi }\right)+\sigma^{2}, \end{array}$$

and

(A39) $$\alpha_{2}=\alpha_{1}{\left(1+\frac{{∥h_{\mathrm{sd}}∥}^{2}\frac{P}{\Phi }}{{∥h_{\mathrm{sd}}∥}^{2}P\left(1-\frac{1}{\Phi }\right)+\sigma^{2}}\right)}^{-1}.$$

At time instant n(3≤n≤N), $\varepsilon_{n}=\varepsilon_{n-1}-\frac{E\left[\varepsilon_{n-1}I_{n}^{H}\right]}{E\left[I_{n}I_{n}^{H}\right]}I_{n}$, where

(A40) $$\begin{array}{cc} \; & E\left[\varepsilon_{n-1}I_{n}^{H}\right]=\sqrt{\alpha_{n-1}}\left(∥h_{\mathrm{sd}}∥\sqrt{\frac{P}{\Phi }}+∥h_{\mathrm{sr}}∥\cdot \left|h_{rd}\right|\beta \sqrt{P}\rho_{n-1}\right),\\ \; & E\left[I_{n}I_{n}^{H}\right]={\left(∥h_{\mathrm{sd}}∥\sqrt{\frac{P}{\Phi }}+∥h_{\mathrm{sr}}∥\cdot \left|h_{rd}\right|\beta \sqrt{P}\rho_{n-1}\right)}^{2}\\ \; & +{∥h_{\mathrm{sr}}∥}^{2}{\left|h_{rd}\right|}^{2}\beta^{2}P\left(\left(1-\frac{1}{\Phi }\right)-{\left(1-\frac{1}{A_{n-1}}\right)}^{2}\rho_{n-1}^{2}\right)+F, \end{array}$$

where $F={∥h_{\mathrm{sd}}∥}^{2}P\left(1-\frac{1}{\Phi }\right)+{\left|h_{rd}\right|}^{2}\beta^{2}\sigma_{r}^{2}+\sigma^{2}$.

So, at time instant *N*, the variance of the final estimation error $\varepsilon_{N}$ is as follows

(A41) $$\alpha_{N}=\alpha_{2}{\left(1+\frac{{\left(∥h_{\mathrm{sd}}∥\sqrt{\frac{P}{\Phi }}+∥h_{\mathrm{sr}}∥\cdot \left|h_{rd}\right|\beta \sqrt{P}\rho^{*}\right)}^{2}}{\Gamma }\right)}^{2-N},$$

where

(A42) $$\Gamma ={∥h_{\mathrm{sr}}∥}^{2}{\left|h_{rd}\right|}^{2}\beta^{2}P\left(\left(1-\frac{1}{\Phi }\right)-{\left(1-\frac{1}{A\left(\rho^{*}\right)}\right)}^{2}{\left(\rho^{*}\right)}^{2}\right)+F,$$

$\alpha_{2}$ is given in (A39). $\rho^{*}$ is defined as the unique solution within [0,1] of the following equation,

(A43) $$\begin{array}{cc} \; & \rho^{2}=\frac{{\left(A\left(\rho \right)\right)}^{2}}{\Phi }\times \\ \; & {\left(1+\frac{{\left(∥h_{\mathrm{sd}}∥\sqrt{\frac{P}{\Phi }}+∥h_{\mathrm{sr}}∥\cdot \left|h_{rd}\right|\beta \sqrt{P}\rho \right)}^{2}}{{∥h_{\mathrm{sr}}∥}^{2}{\left|h_{rd}\right|}^{2}\beta^{2}P\left(\left(1-\frac{1}{\varphi }\right)-{\left(1-\frac{1}{A\left(\rho \right)}\right)}^{2}\rho^{2}\right)+F}\right)}^{-1}, \end{array}$$

and

(A44) $$A\left(\rho \right)=1+\frac{P∥h_{\mathrm{sd}}∥\left(∥h_{\mathrm{sd}}∥\sqrt{\frac{1}{\Phi }}+∥h_{\mathrm{sr}}∥\cdot \left|h_{rd}\right|\beta \rho \right)\left(\sqrt{\frac{1}{\Phi }}-\sqrt{\Phi }\right)}{{∥h_{\mathrm{sr}}∥}^{2}{\left|h_{rd}\right|}^{2}\beta^{2}P\left(\left(1-\frac{1}{\Phi }\right)-{\left(1-\frac{1}{A\left(\rho \right)}\right)}^{2}\rho^{2}\right)+F}.$$

Next, according to (A32) and (A33), we have

(A45) $$\begin{array}{cc} \mathrm{Pr}\left({\tilde{C}}_{N}\right)\leq & \mathrm{Pr}\left\{\varepsilon_{R,N}\notin \left[-\frac{0.5}{2^{\frac{NR\left(N,\epsilon \right)}{2}}},\frac{0.5}{2^{\frac{NR\left(N,\epsilon \right)}{2}}}\right)\right\}+\\ \; & \mathrm{Pr}\left\{\varepsilon_{I,N}\notin \left[-\frac{0.5}{2^{\frac{NR\left(N,\epsilon \right)}{2}}},\frac{0.5}{2^{\frac{NR\left(N,\epsilon \right)}{2}}}\right)\right\}\\ = & 2\mathrm{Pr}\left\{\varepsilon_{R,N}\notin \left[-\frac{0.5}{2^{\frac{NR\left(N,\epsilon \right)}{2}}},\frac{0.5}{2^{\frac{NR\left(N,\epsilon \right)}{2}}}\right)\right\}\\ = & 4Q\left(\frac{0.5}{2^{NR\left(N,\epsilon \right)}\sqrt{\frac{\alpha_{N}}{2}}}\right)=\frac{\epsilon }{2}. \end{array}$$

Substituting (A41) and (A42) into (A45), we obtain

(A46) $$R_{\mathrm{FRC}}^{\mathrm{NFb}}\left(N,\epsilon \right)=R_{\mathrm{FRC}}^{*}\left(N,\epsilon \right)-R_{\mathrm{FRC}}^{◊}\left(N,\epsilon \right),$$

where $R_{\mathrm{FRC}}^{*}\left(N,\epsilon \right)$ and $R_{\mathrm{FRC}}^{◊}\left(N,\epsilon \right)$ are, respectively, given in (51) and (52).

## Appendix C. Proof of Theorem 3

For the MISO FRC case, at time *n*, according to (A22) and (62), the signals received by Eavesdropper can be rewritten as

(A47) $$\begin{array}{cc} \; & Z_{n}=g_{\mathrm{se}}\frac{h_{\mathrm{sd}}^{H}}{∥h_{\mathrm{sd}}∥}X_{n}^{{}^{\prime }}+g_{\mathrm{re}}\frac{{h_{rd}}^{H}}{\left|h_{rd}\right|}{X_{r,n}}^{{}^{\prime }}+\eta_{e,n},1\leq n\leq N,\\ \; & Z_{f,n}=g_{\mathrm{de}}X_{f,n}+\eta_{fe,n},1\leq n\leq N-1. \end{array}$$

The proof of Theorem 3 consists of the following two parts (I)–(II).

(I) To analyze the secrecy level $\Delta_{I}=\frac{H(M|Z_{1}^{N},Z_{f,1}^{N-1},h,g)}{H(M)}$, $H(M|Z_{1}^{N},Z_{f,1}^{N-1},h,g)$ can be bounded by
  (A48) $$\begin{array}{cc} \; & H(M|Z_{1}^{N},Z_{f,1}^{N-1},h,g)\\ \overset{\left(a\right)}{\geq } & H(\Theta |Z_{1}^{N},Z_{f,1}^{N-1},h,g,\eta_{\ell },M_{1}^{N-1})\\ \overset{\left(b\right)}{=} & H(\Theta |\underset{Z_{1}}{\underbrace{\frac{g_{\mathrm{se}}h_{\mathrm{sd}}^{H}}{∥h_{\mathrm{sd}}∥}X_{1}^{{}^{\prime }}+\eta_{e,1}}},\underset{Z_{2}}{\underbrace{\frac{g_{\mathrm{se}}h_{\mathrm{sd}}^{H}}{∥h_{\mathrm{sd}}∥}X_{2}^{{}^{\prime }}+\eta_{e,2}}},\underset{Z_{3}}{\underbrace{\frac{g_{\mathrm{se}}h_{\mathrm{sd}}^{H}}{∥h_{\mathrm{sd}}∥}X_{3}^{{}^{\prime }}+\frac{g_{\mathrm{re}}{h_{rd}}^{H}}{\left|h_{rd}\right|}{X_{r,n}}^{{}^{\prime }}+\eta_{e,3}}},\cdots ,\underset{Z_{N}}{\underbrace{\frac{g_{\mathrm{se}}h_{\mathrm{sd}}^{H}}{∥h_{\mathrm{sd}}∥}X_{N}^{{}^{\prime }}+\frac{g_{\mathrm{re}}{h_{rd}}^{H}}{\left|h_{rd}\right|}{X_{r,N}}^{{}^{\prime }}+\eta_{e,N}}},\\ \; & \underset{Z_{f,1}}{\underbrace{g_{\mathrm{de}}X_{f,1}+\eta_{fe,1}}},\cdots ,\underset{Z_{f,N-1}}{\underbrace{g_{\mathrm{de}}X_{f,N-1}+\eta_{fe,N-1}}},h,g,\eta_{\ell },M_{1}^{N-1})\\ \overset{\left(c\right)}{=} & H(\Theta |\frac{g_{\mathrm{se}}h_{\mathrm{sd}}^{H}}{∥h_{\mathrm{sd}}∥}X_{1}^{{}^{\prime }}+\eta_{e,1})\\ \overset{\left(d\right)}{=} & H\left(\Theta \right)+h\left(\eta_{e,1}\right)-h\left(\frac{g_{\mathrm{se}}h_{\mathrm{sd}}^{H}}{∥h_{\mathrm{sd}}∥}X_{1}^{{}^{\prime }}+\eta_{e,1}\right)\\ \overset{\left(e\right)}{\geq } & H\left(\Theta \right)-\underset{\mathrm{Information}\;\mathrm{leaked}\;\mathrm{by}\;\mathrm{Source}\;\mathrm{at}\;\mathrm{time}\;1}{\underbrace{\log\left(1+\frac{{∥h_{\mathrm{sd}}\cdot g_{\mathrm{se}}^{H}∥}^{2}P}{\sigma_{e}^{2}}\right)}}, \end{array}$$

where

(a) follows from the fact that Θ is a deterministic function of *M*, $\eta_{\ell }=\left(\eta_{1}^{N},\eta_{r,1}^{N},\eta_{f,1}^{N-1},\eta_{e,2}^{N},\eta_{fe,1}^{N-1}\right)$, $M_{1}^{N-1}=\left(M_{\Lambda }\left[\gamma_{1}{\hat{\Theta }}_{1}+V_{1}\right],\cdots ,M_{\Lambda }\left[\gamma_{N-1}{\hat{\Theta }}_{N-1}+V_{N-1}\right]\right)$ and conditioning reduces entropy;
(b) follows from (A47);
(c) follows from $X_{r,1}^{{}^{\prime }}=X_{r,2}^{{}^{\prime }}=0$, $X_{n}^{{}^{\prime }}\left(2\leq n\leq N\right)$ and ${X_{r,n}}^{{}^{\prime }}\left(3\leq n\leq N\right)$ are deterministic functions of $\left(\eta_{n},\eta_{r,n},\eta_{f,n},h\right)$. Additionally, $M_{n}\left(1\leq n\leq N-1\right)$ is only dependent on the complex dither signal sequence $V_{1}^{N-1}$ (see Property (2) of Proposition 2), and $\left(h,g,\eta_{\ell },M_{1}^{N-1}\right)$ is independent of $\left(\Theta ,\eta_{e,1}\right)$;
(d) follows from the fact that Θ is independent of $\eta_{e,1}$;
(e) follows from
  (A49) $$\begin{array}{cc} \; & h\left(\frac{g_{\mathrm{se}}h_{\mathrm{sd}}^{H}}{\left|h_{\mathrm{sd}}\right|}X_{1}^{{}^{\prime }}+\eta_{e,1}\right)-h\left(\eta_{e,1}\right)\\ \leq & \log\det\;\left(\;\pi e\;\left(\;\frac{g_{\mathrm{se}}h_{\mathrm{sd}}^{H}}{∥h_{\mathrm{sd}}∥}X_{1}^{{}^{\prime }}+\eta_{e,1}\;\right)\;{\left(\;\frac{g_{\mathrm{se}}h_{\mathrm{sd}}^{H}}{∥h_{\mathrm{sd}}∥}X_{1}^{{}^{\prime }}+\eta_{e,1}\;\right)}^{H}\;\right)-\log\det\left(\pi e\sigma_{e}^{2}E_{D}\right)\\ = & \log\det\left(\pi e\left(\frac{g_{\mathrm{se}}h_{\mathrm{sd}}^{H}h_{\mathrm{sd}}g_{\mathrm{se}}^{H}}{∥h_{\mathrm{sd}}∥}P+\sigma_{e}^{2}E_{D}\right)\right)-\log\det\left(\pi e\sigma_{e}^{2}E_{D}\right)\\ \overset{\left(e_{1}\right)}{=} & \log\det\left(1+\frac{h_{\mathrm{sd}}g_{\mathrm{se}}^{H}g_{\mathrm{se}}h_{\mathrm{sd}}^{H}}{∥h_{\mathrm{sd}}∥\sigma_{e}^{2}}P\right)=log\left(1+\frac{{∥h_{\mathrm{sd}}g_{\mathrm{se}}^{H}∥}^{2}P}{∥h_{\mathrm{sd}}∥\sigma_{e}^{2}}\right), \end{array}$$
  where $\left(e_{1}\right)$ follows from logdet(AB+E)=logdet(BA+E) and $E_{D}$ represents a D×D identity matrix.
  Substituting (A48) into (64), we conclude that
  (A50) $$\begin{array}{c} \Delta_{I}\geq 1-\frac{\overset{\mathrm{Information}\;\mathrm{leaked}\;\mathrm{by}\;\mathrm{Source}\;\mathrm{at}\;\mathrm{time}\;1}{\overbrace{\log\left(1+\frac{{∥h_{\mathrm{sd}}g_{\mathrm{se}}^{H}∥}^{2}P}{∥h_{\mathrm{sd}}∥\sigma_{e}^{2}}\right)}}}{NR_{\mathrm{FRC}}^{\mathrm{NFb}}\left(N,\epsilon \right)} \end{array}$$
  where $R_{\mathrm{FRC}}^{\mathrm{NFb}}\left(N,\epsilon \right)$ is given in (50).
(II) To analyze secrecy level $\Delta_{II}=\frac{H(M|Z_{1}^{N},Z_{f,1}^{N-1},V_{1}^{N-1},h,g)}{H(M)}$, $H(M|Z_{1}^{N},Z_{f,1}^{N-1},V_{1}^{N-1},h,g)$ can be bounded by
  (A51) $$\begin{array}{cc} \; & H(M|Z_{1}^{N},Z_{f,1}^{N-1},V_{1}^{N-1},h,g)\\ \overset{\left(a\right)}{\geq } & H(\Theta |Z_{1}^{N},Z_{f,1}^{N-1},V_{1}^{N-1},h,g,\eta_{\hbar })\\ \overset{\left(b\right)}{=} & H(\Theta |\underset{Z_{1}}{\underbrace{\frac{g_{\mathrm{se}}h_{\mathrm{sd}}^{H}}{∥h_{\mathrm{sd}}∥}X_{1}^{{}^{\prime }}+\eta_{e,1}}},\underset{Z_{2}}{\underbrace{\frac{g_{\mathrm{se}}h_{\mathrm{sd}}^{H}}{∥h_{\mathrm{sd}}∥}X_{2}^{{}^{\prime }}+\eta_{e,2}}},\underset{Z_{3}}{\underbrace{\frac{g_{\mathrm{se}}h_{\mathrm{sd}}^{H}}{∥h_{\mathrm{sd}}∥}X_{3}^{{}^{\prime }}+\frac{g_{\mathrm{re}}{h_{rd}}^{H}}{\left|h_{rd}\right|}{X_{r,n}}^{{}^{\prime }}+\eta_{e,3}}},\cdots ,\\ \; & \underset{Z_{N}}{\underbrace{\frac{g_{\mathrm{se}}h_{\mathrm{sd}}^{H}}{∥h_{\mathrm{sd}}∥}X_{N}^{{}^{\prime }}+\frac{g_{\mathrm{re}}{h_{rd}}^{H}}{\left|h_{rd}\right|}{X_{r,N}}^{{}^{\prime }}+\eta_{e,N}}},Z_{f,1}^{N-1},V_{1}^{N-1},h,g,\eta_{\hbar })\\ \overset{\left(c\right)}{=} & H(\Theta |\frac{g_{\mathrm{se}}h_{\mathrm{sd}}^{H}}{∥h_{\mathrm{sd}}∥}X_{1}^{{}^{\prime }}+\eta_{e,1},\eta_{e,2}^{N},Z_{f,1}^{N-1},V_{1}^{N-1},h,g,\eta_{\hbar })\\ \overset{\left(d\right)}{=} & H\left(\Theta \right)+h\left(\eta_{e,1}\right)-h\left(\frac{g_{\mathrm{se}}h_{\mathrm{sd}}^{H}}{∥h_{\mathrm{sd}}∥}X_{1}^{{}^{\prime }}+\eta_{e,1}\right)+h\left(\eta_{fe,1}^{N-1}\right)-h\left(Z_{f,1}^{N-1}\right)\\ \geq & H\left(\Theta \right)+h\left(\eta_{e,1}\right)-h\left(\frac{g_{\mathrm{se}}h_{\mathrm{sd}}^{H}}{∥h_{\mathrm{sd}}∥}X_{1}^{{}^{\prime }}+\eta_{e,1}\right)+\sum_{n=1}^{N-1}\left(h\left(\eta_{fe,1}\right)-h\left(Z_{f,1}\right)\right)\\ \overset{\left(e\right)}{\geq } & H\left(\Theta \right)-\underset{\mathrm{Information}\;\mathrm{leaked}\;\mathrm{by}\;\mathrm{Source}\;\mathrm{at}\;\mathrm{time}\;1}{\underbrace{\log\left(1+\frac{{∥h_{\mathrm{sd}}\cdot g_{\mathrm{se}}^{H}∥}^{2}P}{\sigma_{e}^{2}}\right)}}-\underset{\mathrm{Information}\;\mathrm{leaked}\;\mathrm{in}\;\mathrm{feedback}\;\mathrm{channel}\;\mathrm{at}\;\mathrm{time}\;1\;\mathrm{to}\;N-1}{\underbrace{\left(N-1\right)\log\left(1+\frac{{∥g_{\mathrm{de}}∥}^{2}P_{f}}{\sigma_{fe}^{2}}\right)}}, \end{array}$$
  where
  (a) follows from the fact that Θ is a deterministic function of *M*, $\eta_{\hbar }=\left(\eta_{1}^{N},\eta_{r,1}^{N},\eta_{f,1}^{N-1}\right)$, and conditioning reduces entropy;
  (b) follows from (A47);
  (c) follows from $X_{r,1}^{{}^{\prime }}=X_{r,2}^{{}^{\prime }}=0$, $X_{n}^{{}^{\prime }}\left(2\leq n\leq N\right)$ and ${X_{r,n}}^{{}^{\prime }}\left(3\leq n\leq N\right)$ are deterministic functions of $\left(\eta_{n},\eta_{r,n},\eta_{f,n},h\right)$;
  (d) follows from the fact that $X_{1}^{{}^{\prime }}$, $\eta_{e,1}$ is independent of $Z_{f,1}^{N-1}$, for n(1≤n≤N−1), $Z_{f,n}=g_{\mathrm{de}}X_{f,n}+\eta_{fe,n}$ and $X_{f,n}=M_{\Lambda }\left[\gamma_{n}{\hat{\Theta }}_{n}+V_{n}\right]$ are both deterministic functions of $\left(\Theta ,V_{1}^{N-1},h,g,\eta_{\hbar }\right)$;
  (e) follows from
    (A52) $$\begin{array}{cc} \; & h\;\left(\;\frac{g_{\mathrm{se}}h_{\mathrm{sd}}^{H}}{\left|h_{\mathrm{sd}}\right|}X_{1}^{{}^{\prime }}+\eta_{e,1}\;\right)\;-\;h\left(\eta_{e,1}\right)\;\overset{\left(e_{1}\right)}{\leq }\;log\;\left(\;1+\frac{{∥h_{\mathrm{sd}}g_{\mathrm{se}}^{H}∥}^{2}P}{∥h_{\mathrm{sd}}∥\sigma_{e}^{2}}\;\right),\\ \; & h\left(g_{\mathrm{de}}X_{f,n}+\eta_{fe,n}\right)-h\left(\eta_{fe,n}\right)\leq log\left(1+\frac{{∥g_{\mathrm{de}}∥}^{2}P_{f}}{\sigma_{fe}^{2}}\right), \end{array}$$
    where $\left(e_{1}\right)$ is similar to (A49).
  Substituting (A51) into (65), we conclude that
  (A53) $$\begin{array}{cc} \Delta_{I}\geq 1 & -\frac{\overset{\mathrm{Information}\;\mathrm{leaked}\;\mathrm{by}\;\mathrm{Source}\;\mathrm{at}\;\mathrm{time}\;1}{\overbrace{\log\left(1+\frac{{∥h_{\mathrm{sd}}g_{\mathrm{se}}^{H}∥}^{2}P}{∥h_{\mathrm{sd}}∥\sigma_{e}^{2}}\right)}}}{NR_{\mathrm{FRC}}^{\mathrm{NFb}}\left(N,\epsilon \right)}-\frac{\overset{\mathrm{Information}\;\mathrm{leaked}\;\mathrm{in}\;\mathrm{feedback}\;\mathrm{channel}\;\mathrm{at}\;\mathrm{time}\;1\;\mathrm{to}\;N-1}{\overbrace{\left(N-1\right)\log\left(1+\frac{{∥g_{\mathrm{de}}∥}^{2}P_{f}}{\sigma_{fe}^{2}}\right)}}}{NR_{\mathrm{FRC}}^{\mathrm{NFb}}\left(N,\epsilon \right)}, \end{array}$$
  where $R_{\mathrm{FRC}}^{\mathrm{NFb}}\left(N,\epsilon \right)$ is given in (50).

According to (I)–(II), the proof of Theorem 3 is completed.
